# Sensory neuron-derived CGRP ameliorates kidney injury and fibrosis via monocytes and macrophages

**DOI:** 10.1038/s41422-026-01308-7

**Published:** 2026-10-23

**Authors:** Lei Wang, Huaikang Li, Ji Feng, Jianwen Chen, Tongyu Jia, Tailai Zhou, Li Wang, Lu Tang, Xu Zhang, Xin Ma

**Affiliations:** 1 Senior Department of Urology, Chinese PLA General Hospital, Beijing, China; 2 Department of Urology, Chinese PLA 96606 Military Hospital, Luoyang, Henan China; 3 Medical School of Chinese PLA, Beijing, China; 4 Senior Department of Nephrology, Chinese PLA General Hospital, State Key Laboratory of Kidney Diseases, Beijing, China

**Keywords:** Innate immunity, Extracellular signalling molecules

## Abstract

Acute kidney injury (AKI) and its progression to chronic kidney disease (CKD) remain major unmet clinical challenges for which effective therapies are lacking. Although sensory neurons are recognized as key regulators of tissue injury and repair via neuroimmune crosstalk, their organization and function within the kidney are poorly understood. Here, we delineate a renal neuroimmune axis in which nociceptive neurons protect against ischemia-reperfusion (I/R)-induced kidney injury. Using whole-organ tissue clearing and three-dimensional imaging, we mapped an extensive network of nociceptive sensory fibers throughout the renal parenchyma. Ablation of TRPV1^+^ nociceptors aggravated renal dysfunction, tubular necrosis, and fibrosis after I/R, whereas chemogenetic activation conferred protection. Mechanistically, the sensory neuropeptide calcitonin gene-related peptide (CGRP), which is upregulated after injury, acts via the CALCRL–RAMP1 receptor complex on monocytes/macrophages, engaging the cAMP-PKA pathway to suppress pro-inflammatory cytokine and chemokine expression, thereby limiting myeloid infiltration and tubular injury. Importantly, we also found reduced renal CGRP abundance in kidneys from individuals with diabetes and older adults, who are at high risk for AKI and CKD, as well as in corresponding mouse models. In these vulnerable settings, exogenous CGRP more effectively reduced I/R-induced kidney injury. Our findings establish a dorsal root ganglia–kidney neuroimmune circuit that restrains I/R-induced renal inflammation and identify CGRP signaling as a potential therapeutic target to mitigate AKI and its progression to CKD, particularly in high-risk diabetic and elderly populations.

## Introduction

Acute kidney injury (AKI) is a life-threatening condition defined by the rapid deterioration of renal function and represents a major global health problem, affecting more than 13.3 million people worldwide each year.^1,2^ AKI markedly prolongs hospitalization, with mortality ranging from ~10% to 80% depending on the patient population, severity of kidney injury, and level and availability of medical care.^1,3^ Survivors face an elevated risk of chronic kidney disease (CKD), particularly older adults and those with diabetes or hypertension.^4,5^ To date, no pharmacological therapy has been approved to prevent or treat AKI or facilitate recovery from it,^4,6^ underscoring the urgent need for novel therapeutic strategies to mitigate its onset and progression to CKD.

Although kidney injury arises from diverse causes, including ischemia-reperfusion (I/R), nephrotoxicity, glomerular disease, and systemic infection, these etiologies converge on a common pathway of tubular injury, inflammation, and regulated cell death.^7,8^ Inflammation is an early and central driver of kidney injury: cytokines produced by innate immune cells amplify tissue damage and shape subsequent adaptive immune responses.^1,9^ This ostensibly reparative program can become self-sustaining, promoting fibrosis and the AKI-to-CKD transition.^9,10^ In recent decades, macrophages, neutrophils, and T cells have been identified as key effectors in renal I/R injury.^11–13^ Accordingly, targeting immune cells and inflammatory mediators remains a major research focus and a promising avenue for the prevention and treatment of kidney injury.^8,14^

Nociceptors, specialized sensory neurons that innervate peripheral tissues, are increasingly recognized not only for their role in pain transduction but also as active regulators of immune responses.^15–17^ Their immunomodulatory functions extend to tissues such as the meninges, lungs, skin, and viscera, where they regulate inflammation, host defense, and tissue repair in a context-dependent manner.^18–20^ For instance, nociceptors limit inflammatory responses in the lungs and joints,^15,16^ and the neuropeptide calcitonin gene-related peptide (CGRP) mediates immunosuppression during sepsis and promotes wound healing through crosstalk with macrophages and neutrophils.^21–23^ Together, these findings establish a broader framework in which TRPV1^+^ nociceptors and CGRP regulate inflammation, fibrosis, and tissue repair across peripheral tissues. The kidney and nervous system engage in bidirectional crosstalk that is essential for homeostasis.^24^ Although prior work has emphasized renal sympathetic nerves and the cholinergic anti-inflammatory pathway,^24–26^ the anatomical organization, segmental origin, and functional relevance of nociceptive sensory innervation within the renal parenchyma remain poorly defined. Current evidence indicates that renal sensory fibers chiefly target the collecting system and act mainly as mechano- and chemoreceptors; yet, despite the broad immunomodulatory roles of nociceptors in other organs, the existence and physiological relevance of dedicated renal nociceptor populations remain uncertain. Whether this emerging neuroimmune framework extends to the kidney and shapes inflammatory responses during kidney injury is unknown.

Building on this framework, we hypothesized that renal sensory neurons modulate immune responses during kidney injury. Using whole-tissue clearing, we delineated the intact three-dimensional (3D) architecture of nociceptive sensory fibers in the kidney. Functionally, these renal nociceptors protect against renal I/R-induced AKI and mitigate its progression to CKD. Mechanistically, the sensory neuropeptide CGRP signals through the CALCRL–RAMP1 receptor complex on monocytes/macrophages to suppress pro-inflammatory cytokine and chemokine expression, thereby attenuating renal inflammation and tubular damage. Notably, we identified sensory neuropathy in kidneys from individuals with diabetes and older adults, who are at high risk for AKI and CKD, as well as in corresponding mouse models. In these aged and diabetic mice, exogenous CGRP supplementation significantly reduced inflammatory cell infiltration and kidney injury after I/R. Collectively, our findings extend the emerging framework of nociceptor-mediated immune regulation to the kidney by defining the anatomical organization of renal nociceptors, their functional relevance to AKI-to-CKD progression, and the underlying CGRP-myeloid signaling mechanism.

## Results

### Renal nociceptors form an extensive parenchymal network

**Fig. 1:**
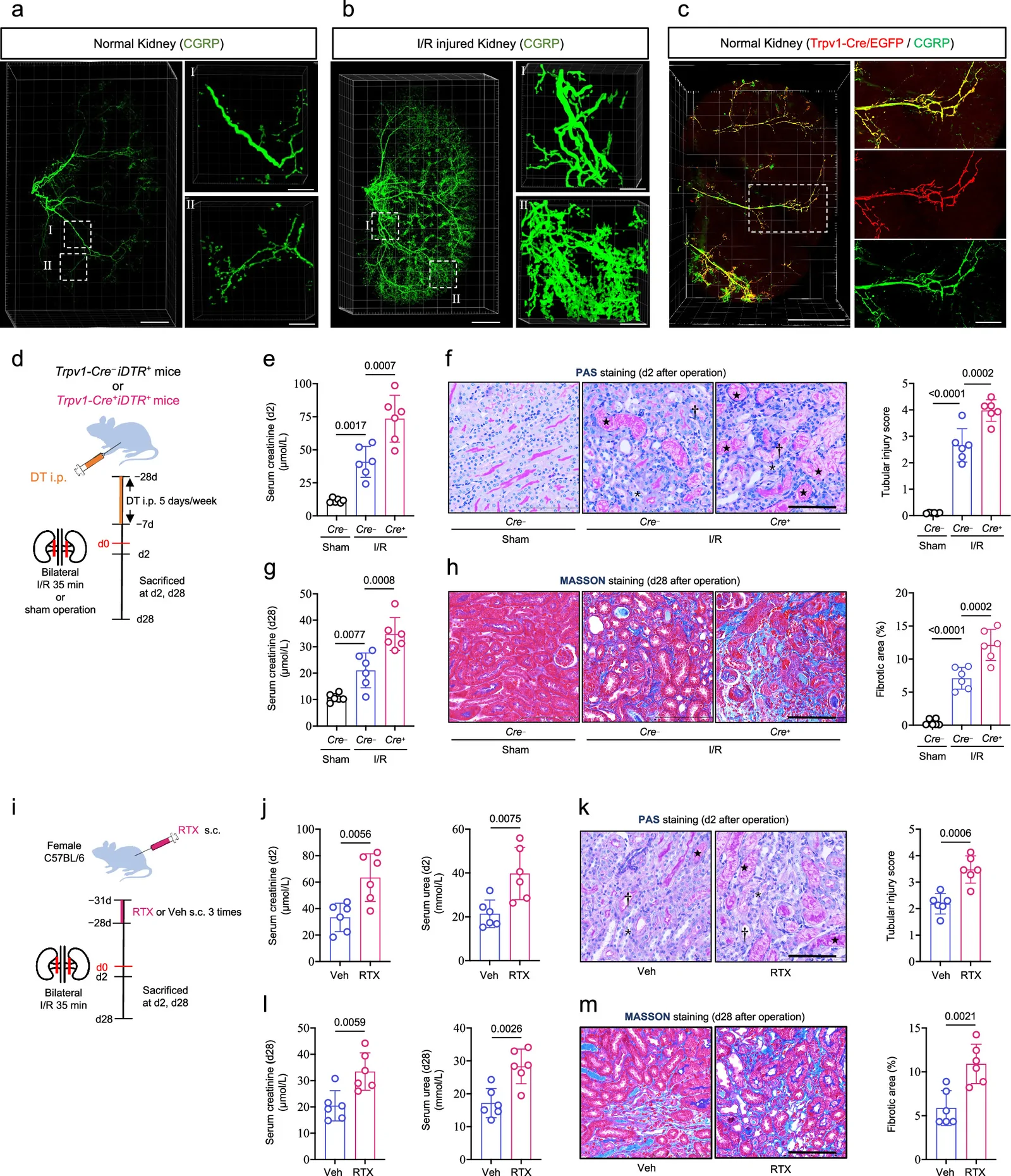
Nociceptive innervation of the kidney and its role in limiting I/R-induced injury. **a**, **b** Representative 3D light-sheet reconstructions of nociceptive fibers (CGRP, green) in SHANEL-cleared whole kidneys: **a** sham surgery; **b** day 2 after I/R. Scale bars, 2 mm (left, overview images) and 200 μm (right, magnified images). **c** 3D reconstructions of TRPV1-EGFP^+^ (red) and CGRP^+^ (green) fibers in CUBIC-cleared kidneys from *Trpv1-Cre*^*+*^*iEGFP*^*+*^ mice. Scale bars, 100 μm. **d** Schematic of DT-mediated ablation of TRPV1^+^ neurons and assessment of AKI (day 2) and CKD (day 28). Initially, 13 *Trpv1-Cre*^+^*iDTR*^+^ mice and 12 *iDTR*^+^ control mice were subjected to I/R, and 12 *iDTR*^+^ mice underwent sham surgery. One *Trpv1-Cre*^+^*iDTR*^+^ mouse died during the acute phase, whereas no deaths occurred in either control group. The surviving mice were then randomly assigned to the AKI and CKD endpoints, with six mice per group at each endpoint. **e**, **g** Serum creatinine levels on day 2 (**e**) and day 28 (**g**) after I/R in DT-treated *Trpv1-Cre*^*+*^*iDTR*^*+*^ mice and *iDTR*^*+*^ controls described in **d**. **f** Representative periodic acid–Schiff (PAS) images on day 2 after I/R (left) and tubular injury scores (right) for the mice in **d**. Tubular injury scoring incorporated the grade of necrosis, casts (★), dilation (†), and brush-border loss (*), with bins corresponding to the percentage of tubules affected: 0 = 0%; 1 = 1%–10%; 2 = 11%–25%; 3 = 26%–45%; 4 = 46%–75%; 5 = 76%–100%. Scale bar, 100 μm. **h** Representative Masson’s trichrome images on day 28 after I/R (left) and fibrosis quantification (right). Scale bar, 100 μm. **i** Schematic of RTX pretreatment and I/R. One of 13 RTX-treated mice died on day 3 after I/R, whereas no death occurred among 12 vehicle-treated mice; *n* = 6 surviving mice per group at each endpoint. **j**, **l** Serum creatinine and urea levels on day 2 (**j**) and day 28 (**l**) after I/R in RTX and vehicle (Veh)-treated mice described in **i**. **k** Representative PAS images on day 2 after I/R (left) and tubular injury scores (right) for the mice in **i**. Scale bar, 100 μm. **m** Representative Masson’s trichrome images on day 28 after I/R (left) and fibrosis quantification (right). Scale bar, 100 μm. Data are mean ± SEM. Statistical analysis: one-way ANOVA with Dunnett’s multiple comparisons test (**e**–**h**); two-tailed unpaired *t*-tests (**j**–**m**).

Although efferent sympathetic nerves innervate the entire kidney, afferent sensory nerves have long been considered largely confined to the renal pelvis, with their parenchymal distribution incompletely defined.^27–29^ To map nociceptive sensory innervation, we applied small-micelle-mediated human organ efficient clearing and labeling (SHANEL), an approach optimized for dense, blood-rich organs,^30^ to whole kidneys immunolabeled for CGRP (a canonical marker of peptidergic nociceptors) and performed light-sheet imaging. Beyond the densely innervated pelvis, CGRP^+^ fibers extensively arborized throughout the renal parenchyma, forming a complex 3D network (Fig. 1a; Supplementary information, Video S1). After renal I/R injury, nociceptor terminals developed cloud-like swellings and exhibited altered morphology (Fig. 1b; Supplementary information, Video S2). To corroborate parenchymal innervation genetically, we crossed *Trpv1-Cre* mice with *iEGFP* reporter mice to label TRPV1^+^ nociceptors with enhanced green fluorescent protein (EGFP). After tissue clearing using clear, unobstructed brain/body imaging cocktails and computational analysis (CUBIC), which preserves fluorescent signals, and subsequent light-sheet imaging, TRPV1-EGFP^+^ fibers co-expressing CGRP were readily detected within the kidney (Fig. 1c). These data reveal extensive nociceptive sensory innervation of the renal parenchyma and raise the possibility that sensory neuronal signaling contributes to kidney pathology.

### Nociceptor elimination exacerbates I/R-induced AKI and subsequent CKD

To assess the functional role of renal nociceptors, we generated *Trpv1-Cre*^*+*^*iDTR*^*+*^ mice and ablated TRPV1^+^ neurons with diphtheria toxin (DT) (Fig. 1d). DT efficiently eliminated TRPV1^+^ neurons in dorsal root ganglia (DRGs) and nodose ganglia (Supplementary information Fig. S1a, b) and eliminated renal CGRP^+^ fibers without altering baseline kidney function, histology, or sympathetic innervation (Supplementary information Fig. S1c–i). Mice were then subjected to 35 min of bilateral renal I/R. Compared with *iDTR*^+^ controls, DT-treated *Trpv1-Cre*^*+*^*iDTR*^*+*^ mice exhibited more severe acute and chronic injury. Serum creatinine and urea levels were higher on day 2 after I/R, accompanied by more severe acute tubular injury, characterized by greater tubular necrosis, luminal dilation, and cast formation (Fig. 1e, f; Supplementary information Fig. S2a). On day 28, renal dysfunction persisted, with increased interstitial collagen deposition detected by Masson staining (Fig. 1g, h; Supplementary information Fig. S2b) and collagen I immunohistochemistry (Supplementary information Fig. S2c), indicating progressive fibrosis. Exploratory experiments in male mice showed high mortality after bilateral I/R, particularly following TRPV1^+^ nociceptor ablation, leaving too few survivors for an adequately powered analysis (Supplementary information Fig. S2d–f). Therefore, the formal mechanistic and longitudinal experiments were conducted on female mice, and the corresponding conclusions are restricted to females.

As an independent strategy, we used resiniferatoxin (RTX), a potent TRPV1 agonist that selectively ablates TRPV1^+^ neurons.^31,32^ Pretreatment with RTX reduced renal CGRP without affecting norepinephrine (NE), baseline kidney function, or histology (Supplementary information Fig. S1j–m), confirming selective sensory depletion. Four weeks later, bilateral I/R produced significantly worse acute and chronic outcomes than those observed in vehicle-treated controls, with impaired renal function and exacerbated histopathology at both days 2 and 28 after I/R (Fig. 1i–m). Together, these independent genetic and pharmacological approaches demonstrate that systemic loss of TRPV1^+^ nociceptors exacerbates I/R-induced AKI and its progression to CKD.

### The kidney receives nociceptive innervation from ipsilateral T10–L2 DRGs

**Fig. 2:**
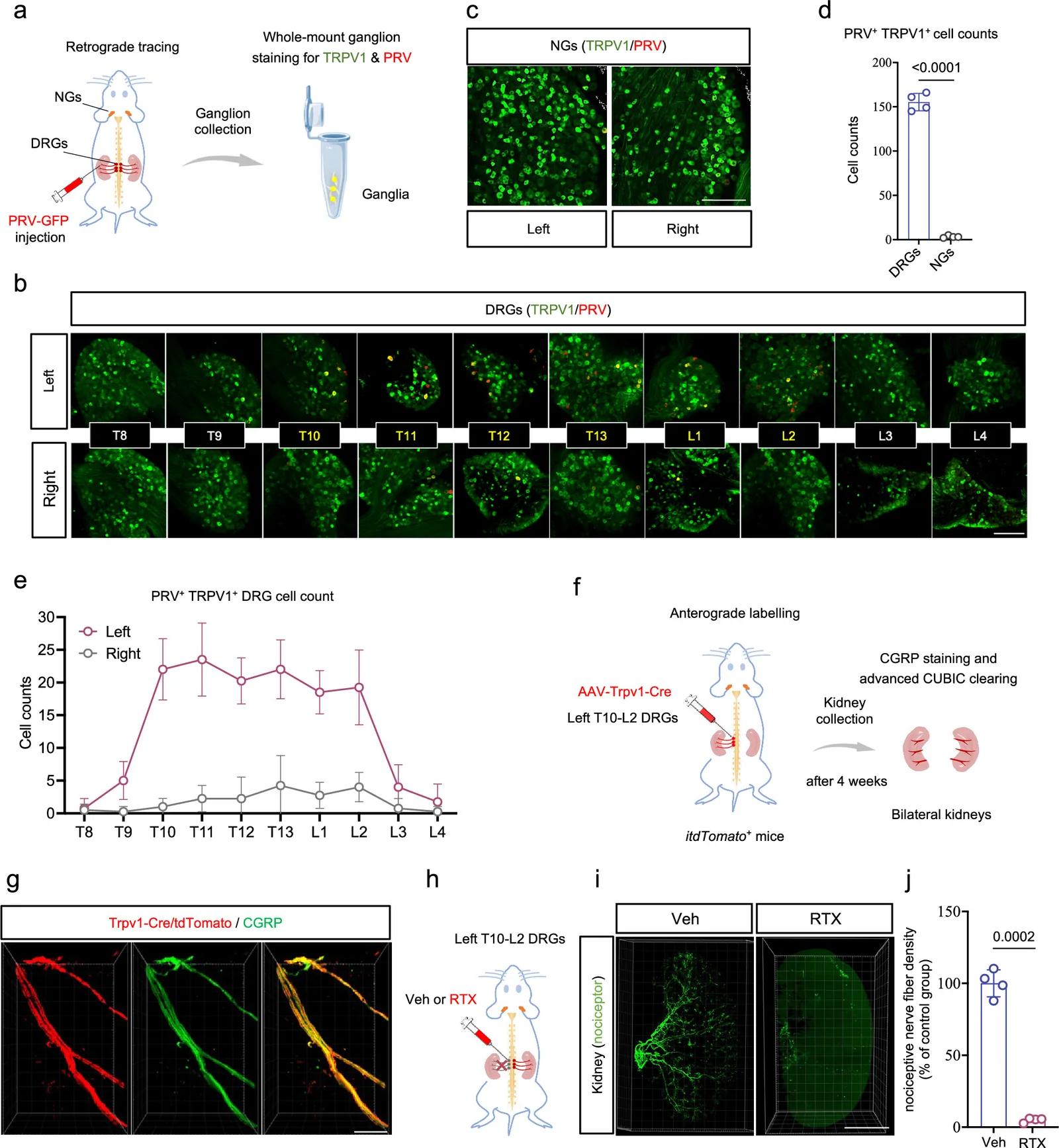
Nociceptor map of the left kidney: projections from T10–L2 DRGs. **a** Schematic of retrograde tracing to identify the cell bodies of nociceptors innervating the left kidney in DRGs or NGs by combining intrarenal PRV-GFP injection with whole-mount anti-TRPV1 immunostaining. *n* = 4 mice. **b**, **c** Representative optical sections of PRV (red)-labeled ganglia from T8–L4 DRGs (**b**) and bilateral NGs (**c**) stained for TRPV1 (green). Scale bar, 200 μm. **d**, **e** Quantification of PRV^+^TRPV1^+^ kidney-innervating nociceptors across DRGs and NGs (**d**) and in the left and right T8–L4 DRGs (**e**). **f** Schematic of anterograde labeling of nociceptor projections from the left T10–L2 DRGs to the left kidney: tdTomato reporter mice received AAV9-Trpv1-Cre, followed by anti-CGRP staining and advanced CUBIC clearing. **g** Representative 3D light-sheet reconstructions showing TRPV1-tdTomato^+^ fibers (red) and CGRP^+^ fibers (green) in CUBIC-cleared kidneys. Scale bar, 100 μm. **h** Schematic of targeted nociceptor ablation in the left T10–L2 DRGs by intraganglionic injection of Veh or RTX in wild-type mice. *n* = 4 mice per group. **i**, **j** Representative 3D light-sheet images (**i**) and quantification (**j**) of CGRP^+^ nociceptive fibers in SHANEL-cleared whole kidneys from Veh- or RTX-treated mice. Scale bar, 2 mm. Data are mean ± SEM. Statistical analysis: two-tailed unpaired *t*-test with Welch’s correction. Sample sizes: *n* = 4 mice per group (**a**–**e**) ; *n* = 4 mice per group (**h**–**j**).

To trace the origin of renal nociceptive fibers, we combined intrarenal injection of the retrograde tracer pseudorabies virus (PRV) with anti-TRPV1 immunostaining in DRGs and nodose ganglia (NGs) (Fig. 2a). PRV^+^TRPV1^+^ neurons innervating the left kidney were predominantly located in DRGs rather than NGs on either side (Fig. 2b, c). Notably, cell bodies were concentrated in the left T10–L2 DRGs ipsilateral to the kidney, with only sparse labeling in adjacent (T8–T9, L3–L4) and contralateral DRGs (Fig. 2d, e), indicating that most nociceptors innervating the left kidney originate in ipsilateral T10–L2 DRGs.

We next validated these findings using anterograde tracing. AAV9-*Trpv1*-Cre was microinjected into the left T10–L2 DRGs of tdTomato reporter mice, followed by CUBIC clearing and 3D imaging. This approach revealed abundant TRPV1-tdTomato^+^ fibers co-expressing CGRP within the left kidney (Fig. 2f, g), whereas essentially no TRPV1‑tdTomato^+^ fibers co‑expressing CGRP were detected in the right kidney (Supplementary information Fig. S3a), confirming direct projections from ipsilateral T10–L2 nociceptors. To test functional specificity, we selectively ablated nociceptors in the left T10–L2 DRGs by local RTX delivery. Consistent with targeted depletion, RTX markedly reduced the density of renal CGRP^+^ fibers on the injected side (Fig. 2h–j).

Prior retrograde tracing using fluorescent cholera toxin subunit B suggested that sensory input to the right kidney similarly originates from the right T10–L2 segments, although quantitative validation was lacking.^33^ Mirroring our left-sided experiments, anterograde AAV9-*Trpv1*-Cre tracing from the right T10–L2 DRGs labeled dense TRPV1-tdTomato^+^CGRP^+^ fibers in the right kidney (Supplementary information Fig. S3b, c), and local RTX administration to these DRGs significantly reduced CGRP^+^ fiber density (Supplementary information Fig. S3d–f). Together, these complementary retrograde and anterograde approaches establish that each kidney receives robust, spatially segregated nociceptive innervation primarily from the ipsilateral T10–L2 DRGs. We next examined whether targeted manipulation of this anatomically defined renal sensory circuit alters acute and chronic kidney injury.

### T10–L2 DRG and local renal nociceptors modulate renal I/R injury

**Fig. 3:**
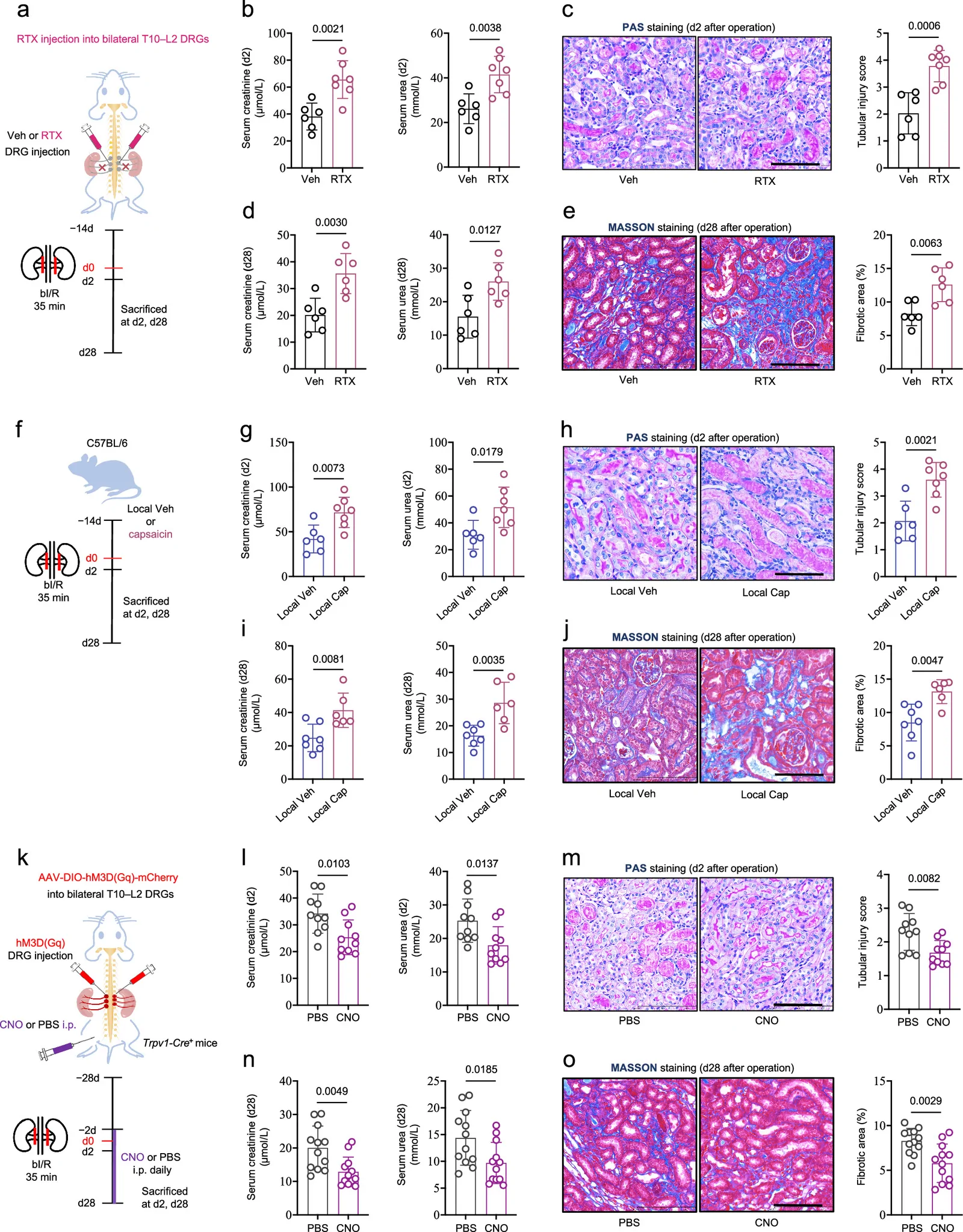
Local and DRG-targeted manipulation of renal nociceptors regulates AKI and AKI-to-CKD progression. **a** Schematic of RTX-mediated ablation of nociceptors in bilateral T10–L2 DRGs before renal I/R. *n* = 6–7 mice per group; no death occurred. **b**, **d** Serum creatinine and urea levels on day 2 (**b**) and day 28 (**d**) after I/R in RTX- and Veh-treated mice. **c** Representative PAS micrographs on day 2 after I/R (left) and tubular injury scores (right). Scale bar, 100 μm. **e** Representative Masson’s trichrome micrographs on day 28 after I/R (left) and fibrosis quantification (right). Scale bar, 100 μm. **f** Schematic of the experimental design used to examine the effect of local renal nociceptor ablation on I/R-induced AKI and subsequent CKD. *n* = 6–7 mice per group; no death occurred. **g**, **i** Serum creatinine and urea levels on day 2 (**g**) and day 28 (**i**) after I/R in mice that received local administration of capsaicin or Veh. **h** Representative PAS images on day 2 (left) and tubular injury scores (right). Scale bar, 100 μm. **j** Representative Masson’s trichrome images on day 28 (left) and fibrosis quantification (right). Scale bar, 100 μm. **k** Schematic of chemogenetic activation of TRPV1^+^ nociceptors in bilateral T10–L2 DRGs using AAV9-DIO-hM3D(G_q_), followed by CNO administration before renal I/R. *n* = 12 mice per group at each endpoint; no death occurred. **l**, **n** Serum creatinine and urea levels on day 2 (**l**) and day 28 (**n**) after I/R in phosphate-buffered saline (PBS)- and CNO-treated *Trpv1-Cre*^**+**^ mice. **m** Representative PAS micrographs on day 2 after I/R (left) and tubular injury scores (right). Scale bar, 100 μm. **o** Representative Masson’s trichrome micrographs on day 28 after I/R (left) and fibrosis quantification (right). Scale bar, 100 μm. Data are mean ± SEM. Statistical analysis: two-tailed unpaired *t*-tests.

Given the protective effect of TRPV1^+^ sensory neurons and the segmental origin of renal nociceptors, we hypothesized that T10–L2 DRG nociceptors play a central role in regulating renal I/R injury. To test this, we bilaterally injected RTX into T10–L2 DRGs to ablate TRPV1^+^ neurons (Supplementary information Fig. S4a) and assessed the functional contribution of this pathway (Fig. 3a). Consistent with systemic nociceptor ablation, targeted elimination of nociceptors in bilateral T10–L2 DRGs markedly exacerbated both acute and chronic kidney injury after I/R, as evidenced by higher serum creatinine and urea levels on days 2 and 28, more severe acute tubular injury, and increased interstitial fibrosis (Fig. 3b–e).

To determine whether direct disruption of local renal nociceptive input is sufficient to aggravate injury, we applied capsaicin (33 mM) perivascularly around the renal artery (Supplementary information Fig. S4b). This intervention reduced renal CGRP without altering NE, confirming selective impairment of local sensory fibers (Supplementary information Fig. S4c). Consistent with the effects of systemic and DRG-targeted ablation, local capsaicin treatment worsened renal dysfunction and tubular injury on day 2 and exacerbated renal dysfunction and interstitial fibrosis on day 28 after I/R (Fig. 3f–j).

By contrast, ablation of vagal TRPV1^+^ nociceptors by DT injection into the bilateral nodose ganglia of *Trpv1-Cre*^*+*^*iDTR*^*+*^ mice did not significantly alter acute or chronic kidney injury after I/R (Supplementary information Fig. S4d–i), supporting a predominant contribution of DRG-derived rather than vagal sensory input.

Conversely, chemogenetic activation of T10–L2 nociceptors was protective. In *Trpv1*-Cre^+^ mice, bilateral AAV9-DIO-hM3D(G_q_)-mCherry injection into T10–L2 DRGs restricted hM3D(G_q_) expression predominantly to TRPV1^+^ neurons (Supplementary information Fig. S4j). Clozapine N-oxide (CNO) administration increased renal CGRP levels at baseline and after I/R (Supplementary information Fig. S4k), improved acute renal function, reduced tubular injury, and attenuated chronic renal dysfunction and fibrosis (Fig. 3k–o).

Together, these complementary local and DRG-targeted loss- and gain-of-function approaches establish that the renal nociceptive circuit protects against I/R-induced AKI and limits its progression to CKD.

### Nociceptor-derived CGRP mitigates I/R-induced AKI and CKD

**Fig. 4:**
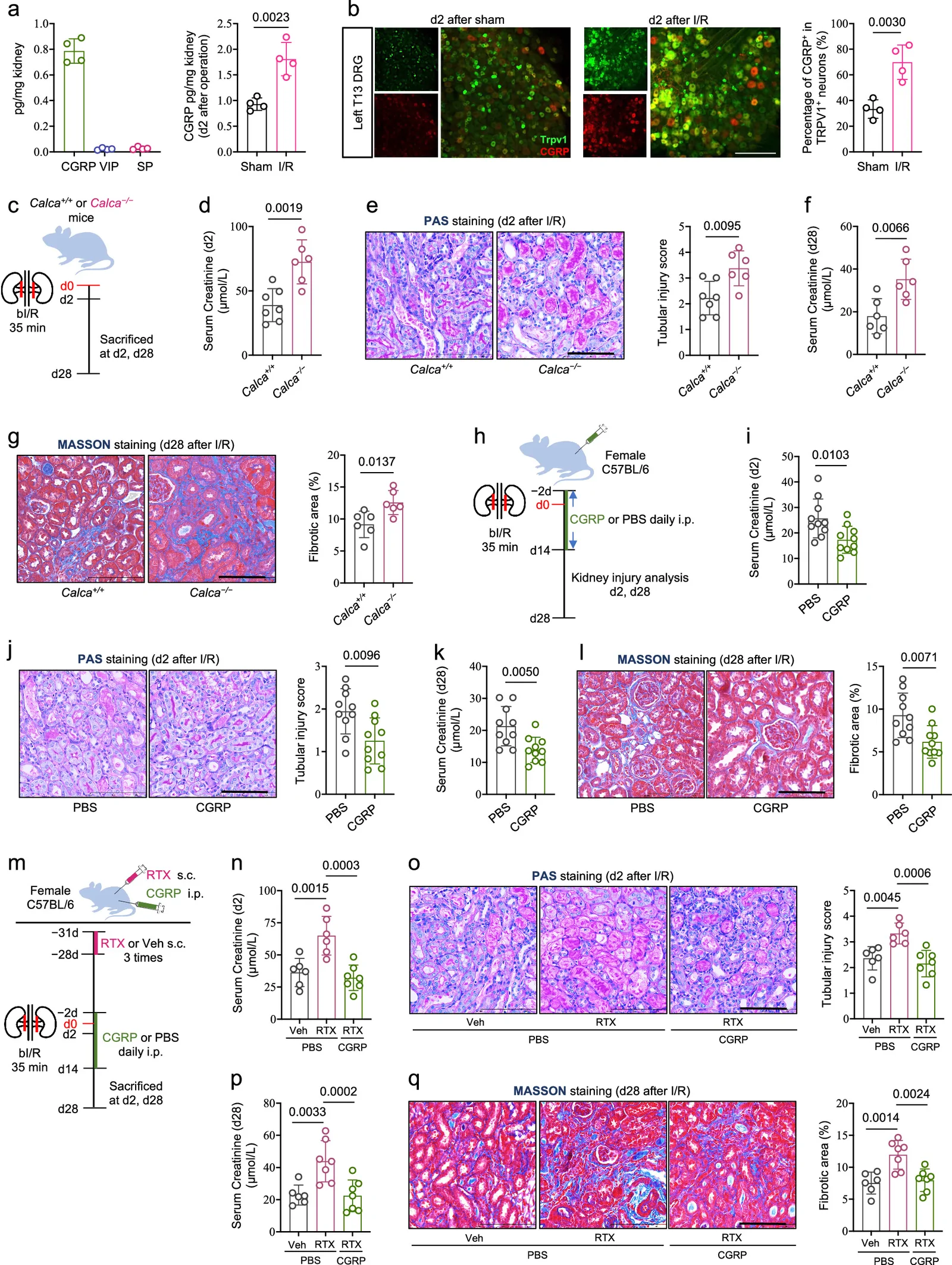
The sensory neuropeptide CGRP modulates renal I/R injury. **a** ELISA measurements of renal neuropeptide levels in 8-week-old female C57BL/6 mice: left, baseline levels of CGRP, VIP, and SP; right, CGRP levels on day 2 after sham surgery or I/R. *n* = 4 mice per group. **b** Representative immunofluorescence images of DRGs on day 2 after sham surgery or renal I/R, showing TRPV1 (green) and CGRP (red), with quantification in the right panel. Scale bar, 200 μm. *n* = 4 mice per group. **c** Schematic of the *Calca* knockout experiment. Of 13 mice initially enrolled per genotype, one *Calca*^*−/−*^ mouse died during the acute phase, whereas no death occurred in the *Calca*^*+/+*^ group; *n* = 6–7 mice per group at each endpoint. **d**, **f** Serum creatinine levels on day 2 (**d**) and day 28 (**f**) after I/R in the *Calca*^*+/+*^ and *Calca*^*−/−*^ mice described in **c**. **e** Representative PAS images on day 2 (left) and tubular injury scores (right) for the mice in **c**. Scale bar, 100 μm. **g** Representative Masson’s trichrome images on day 28 (left) and fibrosis quantification (right). Scale bar, 100 μm. **h** Schematic of exogenous CGRP supplementation (day −2 to day +14 relative to I/R). *n* = 10 mice per group; no death occurred. **i**, **k** Serum creatinine levels on day 2 (**i**) and day 28 (**k**) after I/R in PBS- and CGRP-treated mice. **j** Representative PAS on day 2 (left) and tubular injury scores (right). Scale bar, 100 μm. **l** Representative Masson’s trichrome images on day 28 (left) and fibrosis quantification (right). Scale bar, 100 μm. **m** Schematic of CGRP supplementation in RTX-denervated mice. **n**, **p** Serum creatinine levels on day 2 (**n**) and day 28 (**p**) after I/R in control and RTX-treated mice receiving daily intraperitoneal injections of PBS or CGRP. One of 14 RTX + PBS-treated mice died during the acute phase, whereas no death occurred among 12 Veh + PBS-treated mice or 14 RTX + CGRP-treated mice; *n* = 6–7 mice per group at each endpoint. **o** PAS images and acute tubular injury scores on day 2. Scale bar, 100 μm. **q** Masson’s trichrome images and fibrosis quantification on day 28. Scale bar, 100 μm. Data are mean ± SEM. Statistical analysis: two-tailed unpaired *t*-tests (**b**, **d**–**g**, **i**–**l**); one-way ANOVA with Dunnett’s multiple comparisons test (**n**–**q**).

Because visceral nociceptors release neuropeptides upon tissue damage and modulate injury via neuroimmune regulation,^34,35^ we next explored which mediators link nociceptors to kidney injury. We quantified renal neuropeptides by ELISA and found that, among CGRP, vasoactive intestinal peptide (VIP), and substance P (SP), only CGRP was abundantly expressed at baseline and increased significantly after I/R (Fig. 4a; Supplementary information Fig. S5a). Consistent with this finding, CGRP expression in the cell bodies of kidney-innervating DRG neurons was upregulated after renal I/R injury (Fig. 4b). Together with the increased renal CGRP measured by ELISA, the morphological changes described above are consistent with altered nociceptor activity and may reflect enhanced CGRP signaling after I/R.

To assess whether CGRP mediates the protective effect of nociceptors in kidney injury, we used *Calca*^*−/−*^ mice, which lack CALCA-derived αCGRP. *Calca*^*−/−*^ mice developed more severe acute and chronic injury after I/R than *Calca*^*+/+*^ controls, with impaired renal function and exacerbated histopathology on days 2 and 28 (Fig. 4c–g; Supplementary information Fig. S5b). Conversely, systemic CGRP supplementation (initiated 2 days before I/R and continued for 14 days) ameliorated acute and chronic injury in wild-type mice, as demonstrated by improved renal function and histopathological outcomes on days 2 and 28 after I/R (Fig. 4h–l; Supplementary information Fig. S5c). Furthermore, systemic CGRP supplementation rescued the aggravated injury phenotype caused by RTX-mediated nociceptor ablation (Fig. 4m–q; Supplementary information Fig. S5d). These findings identify DRG-derived CGRP as a key neuropeptide that limits I/R-induced kidney injury.

### CGRP limits injury by modulating monocytes and macrophages via CALCRL–RAMP1

**Fig. 5:**
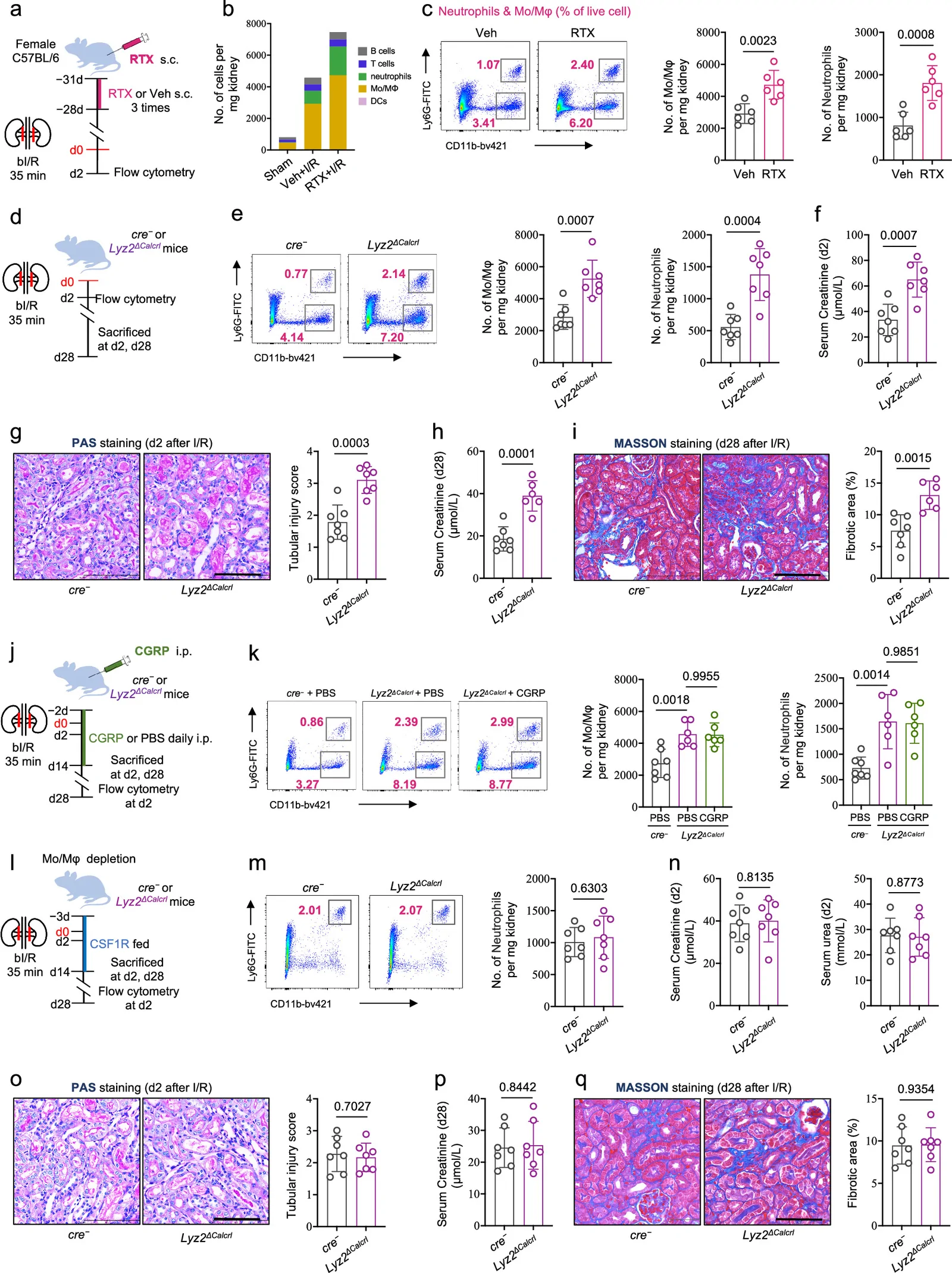
CGRP protects the kidney by modulating monocytes/macrophages. **a** Schematic of inflammatory profiling on day 2 after I/R using samples from the RTX experiment shown in Fig. 1i; *n* = 6 mice per group. **b** Combined quantification of the major renal immune cell subsets analyzed. **c** Representative pseudocolor plots (left) and quantification (right) showing absolute numbers (per mg of kidney tissue) of CD11b^hi^ Ly6G^−^ CD11cˡᵒ monocytes/macrophages (Mo/Mφ) and CD11b^hi^ Ly6G^+^ neutrophils in RTX- and Veh-treated mice on day 2 after I/R. **d** Schematic of myeloid CGRP receptor deletion. Of 14 mice initially enrolled per genotype, one *Lyz2*^*ΔCalcrl*^ mouse died on day 5 after I/R, whereas no death occurred in the control group; *n* = 6–7 mice per group at each endpoint. **e** Representative pseudocolor plots (left) and quantification (right) of renal Mo/Mφ and neutrophils in *Lyz2*^*ΔCalcrl*^ mice and *Calcrl*^*fl/fl*^ controls on day 2 after I/R. **f**, **h** Serum creatinine levels on day 2 (**f**) and day 28 (**h**) after I/R in *Lyz2*^*ΔCalcrl*^ and *Calcrl*^*fl/fl*^ mice. **g** Representative PAS images (left) and tubular injury scores (right) on day 2 after I/R. Scale bar, 100 μm. **i** Representative Masson’s trichrome images (left) and fibrosis quantification (right) on day 28 after I/R. Scale bar, 100 μm. **j** Schematic of CGRP supplementation in mice with myeloid *Calcrl* deletion (*Lyz2*^*ΔCalcrl*^). **k** Representative flow-cytometry plots (left) and quantification (right) of absolute numbers of Mo/Mφ and neutrophils on day 2 after I/R in PBS-treated *Calcrl*^*fl/fl*^ controls and in *Lyz2*^*ΔCalcrl*^ mice treated with CGRP or PBS. **l** Schematic of the experiment designed to determine whether Mo/Mφ are required for the exacerbated injury caused by myeloid *Calcrl* deletion, using a CSF1R inhibitor to reduce Mo/Mφ. *n* = 7 mice per group at each endpoint; no death occurred. **m** Flow cytometric analysis of renal CD11b^hi^ Ly6G^+^ neutrophils on day 2 after I/R: representative plots (left) and quantification (right). **n** Serum creatinine and urea levels on day 2 after I/R in *Lyz2*^*ΔCalcrl*^ and *Calcrl*^*fl/fl*^ mice following CSF1R inhibitor treatment. **o** PAS images and tubular injury scores on day 2. Scale bar, 100 μm. **p** Serum creatinine levels on day 28 after I/R in *Lyz2*^*ΔCalcrl*^ and *Calcrl*^*fl/fl*^ mice following CSF1R inhibitor treatment. **q** Masson’s trichrome images and fibrosis quantification on day 28. Scale bar, 100 μm. Data are mean ± SEM. Statistical analysis: two-tailed unpaired *t*-tests (**c**, **e**–**i**, **m**–**q**); one-way ANOVA with Dunnett’s multiple comparisons test (**k**).

To define how sensory denervation reshapes inflammation after I/R, we performed flow cytometric profiling of renal leukocytes on day 2 after I/R in mice subjected to systemic RTX ablation and in vehicle controls. Nociceptor ablation specifically increased the infiltration of CD11b^hi^ Ly6G^−^ CD11c^lo^ monocytes/macrophages (Mo/Mφ) and CD11b^hi^ Ly6G^+^ neutrophils on day 2 after I/R, whereas the abundance of dendritic cells, B cells, and T cells was unchanged (Fig. 5a–c; Supplementary information Fig. S6a–c). This myeloid-cell accumulation elicited by sensory denervation mirrors prior reports in colitis, cerebral and pulmonary infections, and diverse tissue-injury models.^21,22,31,36^ Notably, exogenous CGRP normalized the RTX-induced increase in Mo/Mφ and neutrophils (Supplementary information Fig. S6d, e). Given that CGRP dampens Mo/Mφ and neutrophil infiltration, and considering the central role of innate immunity in renal I/R injury, we hypothesized that CGRP signals to innate immune cells to attenuate inflammation and tubular injury.

To test this possibility, we generated mice with myeloid-specific deletion of the *Calcrl* gene, which encodes a component of the CGRP receptor, by crossing *Lyz2-Cre* mice with *Calcrl*^*fl/fl*^ mice (hereafter *Lyz2*^*ΔCalcrl*^) (Supplementary information Fig. S7a, left). Relative to *Calcrl*^*fl/fl*^ mice, *Lyz2*^*ΔCalcrl*^ mice showed increased renal infiltration of CD11b^hi^ Ly6G^−^ CD11cˡᵒ Mo/Mφ and CD11b^hi^ Ly6G^+^ neutrophils on day 2 after I/R (Fig. 5d, e). These changes were accompanied by more severe acute and chronic kidney injury than in controls, as evidenced by worsened renal function and exacerbated histopathology on days 2 and 28 (Fig. 5f–i; Supplementary information Fig. S7a, right).

We next asked whether CGRP can protect against renal I/R injury independently of the canonical CALCRL–RAMP1 receptor complex. To test this, we administered CGRP to *Lyz2*^*ΔCalcrl*^ mice and found that exogenous CGRP neither reduced the increased infiltration of Mo/Mφ and neutrophils (Fig. 5j, k) nor rescued the aggravated acute and chronic kidney injury caused by myeloid-specific *Calcrl* deletion (Supplementary information Fig. S7b–f). These data demonstrate that the protective effects of CGRP in renal I/R injury require signaling through the CALCRL–RAMP1 receptor on myeloid cells.

To test whether Mo/Mφ are the principal target population, we reduced renal Mo/Mφ abundance using a CSF1R inhibitor (efficacy confirmed in the kidney after I/R; Supplementary information Fig. S7g–l). Administration of the CSF1R inhibitor abrogated the increases in leukocyte infiltration and acute and chronic kidney injury in *Lyz2*^*ΔCalcrl*^ mice and eliminated the associated neutrophilia (Fig. 5l–q; Supplementary information Fig. S7l), indicating that the enhanced neutrophil recruitment caused by myeloid *Calcrl* deletion is Mo/Mφ dependent.

**Fig. 6:**
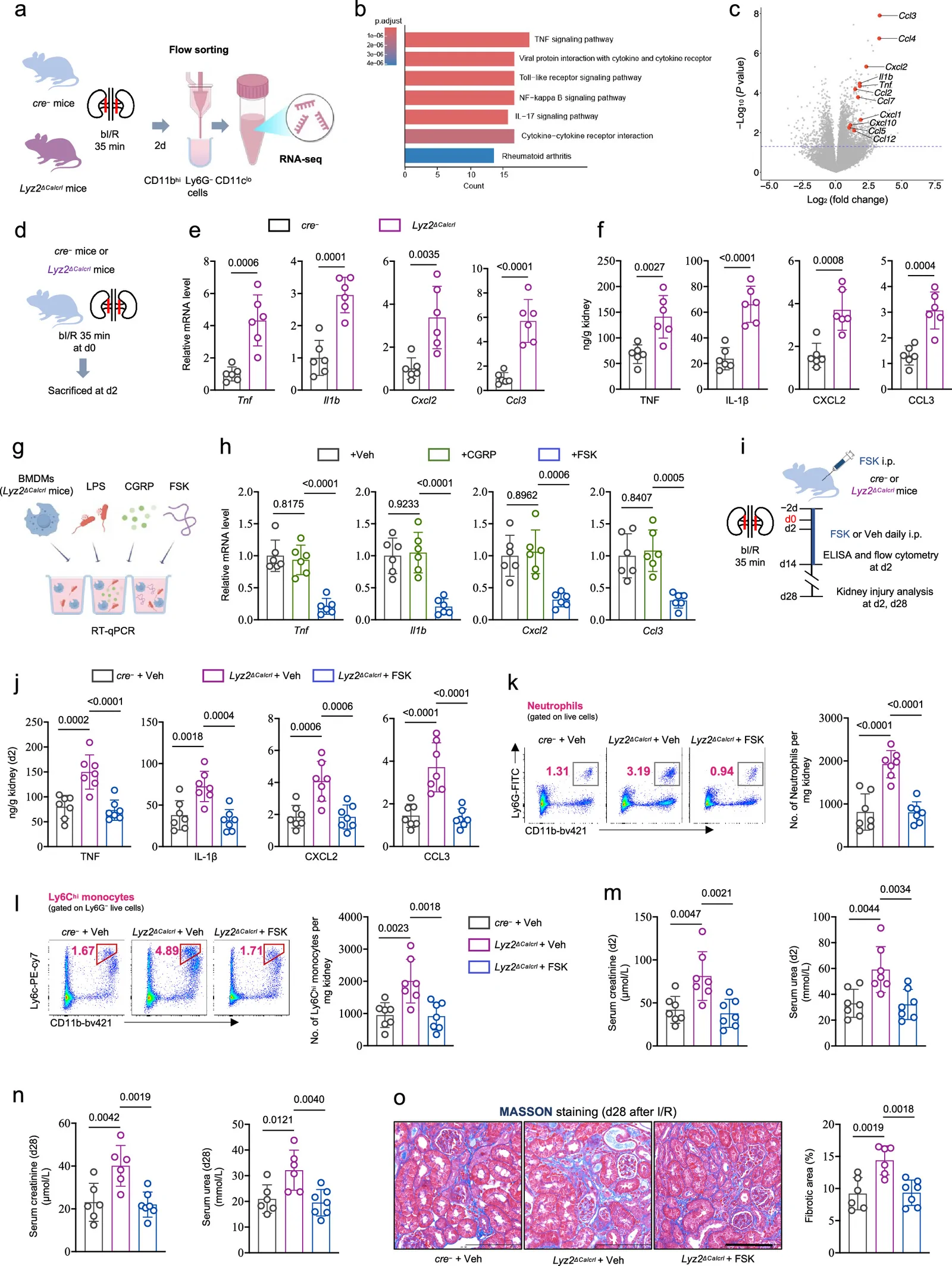
CGRP acts through CALCRL–RAMP1 on mononuclear phagocytes to limit inflammation via cAMP-PKA signaling. **a** Schematic of bulk RNA-seq of renal CD11b^hi^ Ly6G^−^ CD11cˡᵒ monocytes/macrophages (Mo/Mφ) sorted on day 2 after I/R in *Lyz2*^*ΔCalcrl*^ and *Calcrl*^*fl/fl*^ mice. *n* = 5 mice per group. **b** KEGG pathway enrichment analysis of DEGs in Mo/Mφ from *Lyz2*^*ΔCalcrl*^ mice compared with those from *Calcrl*^*fl/fl*^ mice on day 2 after I/R. **c** Volcano plot showing 554 upregulated and 179 downregulated genes in Mo/Mφ from *Lyz2*^*ΔCalcrl*^ mice relative to those from *Calcrl*^*fl/fl*^ mice; chemotaxis- and pro-inflammatory cytokine-related genes are highlighted. **d** Schematic of chemokine/cytokine analyses on day 2 after I/R in *Lyz2*^*ΔCalcrl*^ and *Calcrl*^*fl/fl*^ mice. *n* = 6 mice per group. **e** RT-qPCR analysis of representative pro-inflammatory transcripts (*Tnf*, *Il1b*, *Cxcl2*, and *Ccl3*) in sorted renal CD11b^hi^ Ly6G^−^ CD11cˡᵒ Mo/Mφ on day 2 after I/R. **f** ELISA measurements of the corresponding cytokines and chemokines in whole-kidney lysates from the cohort described in **d**. **g** In vitro experimental design: BMDMs from *Lyz2*^*ΔCalcrl*^ mice were stimulated with LPS (100 ng/mL) ± CGRP or forskolin (FSK); *n* = 6 biological replicates per condition. **h** qPCR analysis of *Tnf*, *Il1b*, *Cxcl2*, and *Ccl3* mRNA levels in the BMDMs from **g**: LPS alone, LPS + CGRP (green), or LPS + FSK (blue). **i** Schematic of the in vivo FSK rescue experiment in *Lyz2*^*ΔCalcrl*^ mice (daily intraperitoneal administration before and after I/R). One of 14 mice in the *Lyz2*^*ΔCalcrl*^ + Veh group died during the acute phase, whereas no death occurred among 13 *Calcrl*^*fl/fl*^ + Veh mice or 14 *Lyz2*^*ΔCalcrl*^ + FSK mice; *n* = 6–7 mice per group at each endpoint. **j** ELISA quantification of TNF, IL-1β, CXCL2, and CCL3 in kidney homogenates on day 2 after I/R. **k** Representative pseudocolor plots (left) and quantification (right) of absolute numbers of neutrophils per mg of kidney tissue in Veh-treated *Calcrl*^*fl/fl*^ controls and *Lyz2*^*ΔCalcrl*^ mice treated with FSK or Veh on day 2 after I/R. **l** Representative pseudocolor plots (left) and quantification (right) of absolute numbers of CD11b^hi^ Ly6C^hi^ classical monocytes per mg of kidney tissue in Veh-treated *Calcrl*^*fl/fl*^ controls and *Lyz2*^*ΔCalcrl*^ mice treated with FSK or Veh on day 2 after I/R. **m**, **n** Serum creatinine and urea levels on day 2 (**m**) and day 28 (**n**) after I/R in *Lyz2*^*ΔCalcrl*^ and *Calcrl*^*fl/fl*^ mice treated with FSK or Veh. **o** Masson’s trichrome images on day 28 and fibrosis quantification. Scale bar, 100 μm. Data are mean ± SEM. Statistical analysis: two-tailed unpaired *t*-tests (**e**, **f**); one-way ANOVA with Dunnett’s multiple comparisons test (**h**–**o**).

To delineate the underlying transcriptional program, we performed bulk RNA sequencing (RNA-seq) of renal CD11b^hi^ Ly6G^−^ CD11cˡᵒ Mo/Mφ purified by a two-step strategy (magnetically activated cell sorting (MACS) enrichment followed by fluorescence-activated cell sorting (FACS)) from *Lyz2*^*ΔCalcrl*^ mice and *Calcrl*^*fl/fl*^ controls on day 2 after I/R (Fig. 6a). In *Lyz2*^*ΔCalcrl*^ Mo/Mφ, pathway analysis of upregulated differentially expressed genes (DEGs) revealed significant enrichment of pro-inflammatory and pro-chemotactic signaling, including TNF, Toll-like receptor, and NF-κB modules (Fig. 6b). Consistent with these results, pro-inflammatory and chemotaxis-related genes, including *Tnf*, *Il1b*, *Cxcl2*, and *Ccl3*, were markedly upregulated in *Lyz2*^*ΔCalcrl*^ Mo/Mφ (Fig. 6c) (BioProject: PRJNA1367940).

Reverse transcription quantitative PCR (RT-qPCR) analysis of sorted renal CD11b^hi^ Ly6G^−^ CD11cˡᵒ Mo/Mφ on day 2 after I/R confirmed significantly elevated expression of genes encoding the pro-inflammatory cytokines *Tnf* and *Il1b*, as well as the chemokines *Cxcl1*, *Cxcl2*, *Cxcl10*, *Ccl2*, *Ccl3*, *Ccl4*, *Ccl5*, and *Ccl7*, in *Lyz2*^*ΔCalcrl*^ mice (Fig. 6d, e; Supplementary information Fig. S8a). This transcriptional upregulation was mirrored at the protein level in whole-kidney lysates, with increases in TNF, IL-1β, CXCL2, and CCL3 (Fig. 6f). Notably, this amplified pro-inflammatory program is consistent with patterns observed after sensory denervation in other tissue-injury settings.^22,31^

We next asked whether CGRP directly reprograms macrophages under inflammatory conditions. Lipopolysaccharide (LPS) was used here as a standardized in vitro inflammatory stimulus for mechanistic analysis, rather than as a surrogate model of renal I/R injury. Bone-marrow-derived macrophages (BMDMs) stimulated with LPS showed upregulated mRNA expression of pro-inflammatory cytokine genes (*Tnf*, *Il1b*) and chemokine genes (*Cxcl2*, *Ccl3*), whereas co-treatment with CGRP suppressed their expression (Supplementary information Fig. S8b, c). Because the CALCRL–RAMP1 complex is a G protein-coupled receptor that signals through Gα_s_ to elevate cAMP and activate PKA,^37^ and because previous work showed that CGRP engages the cAMP-PKA axis to restrain macrophage chemotactic programs,^31^ we tested whether direct activation of this pathway could bypass receptor loss. In BMDMs from *Lyz2*^*ΔCalcrl*^ mice, CGRP failed to suppress LPS-induced expression of *Tnf*, *Il1b*, *Cxcl2*, and *Ccl3*, whereas forskolin, an adenylyl cyclase activator, reduced the abundance of these transcripts despite CGRP receptor loss (Fig. 6g, h).

To determine whether the cAMP pathway mediates the effects of CGRP in vivo, we administered forskolin daily to *Lyz2*^*ΔCalcrl*^ mice before and after renal I/R (Fig. 6i). On day 2, forskolin normalized the increased cytokine/chemokine expression (Fig. 6j) and reduced the abundance of renal neutrophils and CD11b^hi^ Ly6C^hi^ classical monocytes (Fig. 6k, l), two principal inflammatory populations recruited from the circulation after kidney injury in response to chemoattraction by CXCL2 and CCL3, respectively.^38,39^ Correspondingly, forskolin treatment ameliorated both acute and chronic functional impairment and histological damage (Fig. 6m–o; Supplementary information Fig. S8d, e). Together, these data indicate that CGRP signals through CALCRL–RAMP1 on Mo/Mφ to engage the cAMP-PKA pathway, thereby suppressing pro-inflammatory gene programs, limiting leukocyte recruitment, and protecting the kidney.

### CGRP alleviates I/R-induced injury in diabetic and aged mice

**Fig. 7:**
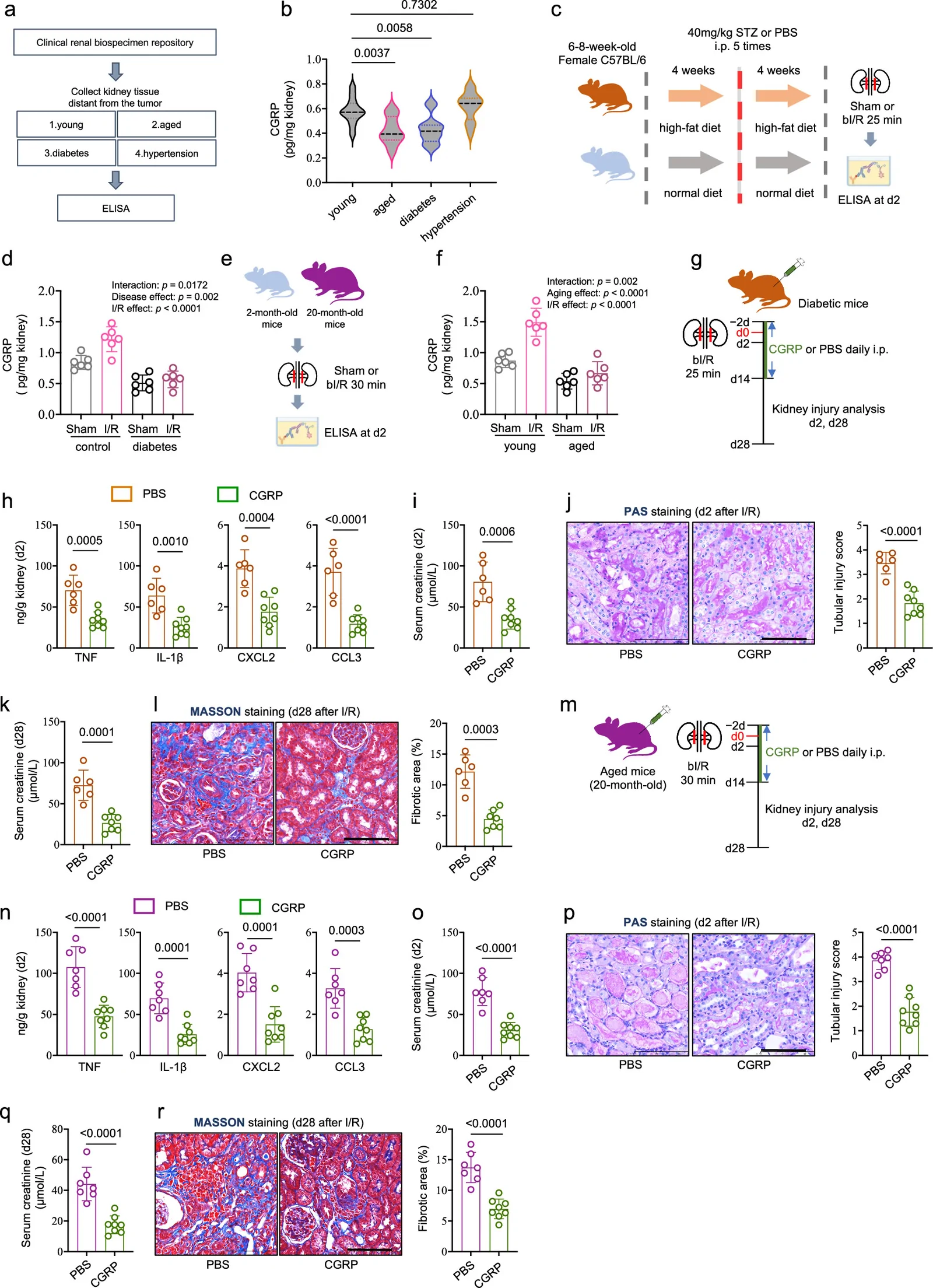
CGRP mitigates acute and chronic I/R injury in diabetic and aged mice. **a**, **b** Schematic of ELISA-based analysis of human nephrectomy samples (**a**) and renal CGRP levels across cohorts (**b**). *n* = 12 patients per group. **c**, **d** Schematic of the diabetic mouse model (**c**) and renal CGRP levels at baseline and on day 2 after I/R in diabetic and control mice (**d**). *n* = 6 mice per group. **e**, **f** Schematic of the aged mouse model (**e**) and renal CGRP levels at baseline and on day 2 after I/R in aged and young mice (**f**). *n* = 6 mice per group. **g** Schematic of exogenous CGRP supplementation in the diabetic I/R model. Of 15 PBS-treated mice initially enrolled, two died before endpoint allocation, and one mouse assigned to the day-28 endpoint subsequently died. No deaths occurred among the 15 CGRP-treated mice; *n* = 6–8 surviving mice per group at each endpoint. **h** ELISA measurements of renal TNF-α, IL-1β, CXCL2, and CCL3 on day 2 after I/R (diabetic model). **i**, **k** Serum creatinine levels on day 2 (**i**) and day 28 (**k**) after I/R in PBS- and CGRP-treated diabetic mice. **j** Representative PAS images (left) and tubular injury scores (right) on day 2 (diabetic model). Scale bar, 100 μm. **l** Representative Masson’s trichrome images (left) and fibrosis quantification (right) on day 28 (diabetic model). Scale bar, 100 μm. **m** Schematic of exogenous CGRP supplementation in the aged I/R model. Two of 16 PBS-treated mice died during the initial phase, whereas no death occurred among 16 CGRP-treated mice; *n* = 7–8 surviving mice per group at each endpoint. **n** ELISA measurements of renal TNF-α, IL-1β, CXCL2, and CCL3 on day 2 after I/R (aged model). **o**, **q** Serum creatinine levels on day 2 (**o**) and day 28 (**q**) after I/R in PBS- and CGRP-treated aged mice. **p** Representative PAS images (left) and tubular injury scores (right) on day 2 (aged model). Scale bar, 100 μm. **r** Representative Masson’s trichrome images (left) and fibrosis quantification (right) on day 28 (aged model). Scale bar, 100 μm. Data are mean ± SEM. Statistical analysis: one-way ANOVA with Dunnett’s multiple comparisons test (**b**); two-way ANOVA with Šidák’s multiple comparisons test (**d**, **f**); two-tailed unpaired *t*-tests (**h**–**l**, **n**–**r**).

Our previous experiments in young, healthy mice (8–10 weeks) established a protective role for CGRP in I/R-induced kidney injury: kidneys are innervated by CGRP^+^ nociceptive fibers, renal CGRP increases after I/R, and this neuroimmune signal limits inflammation via Mo/Mφ. To assess the clinical relevance of these findings, given that severe CKD predominantly affects older individuals or patients with underlying metabolic or circulatory diseases, we next asked whether CGRP^+^ nociceptive signaling is impaired with aging or in the context of chronic disease.^5,40,41^ We quantified renal CGRP levels by ELISA in a clinical biospecimen cohort comprising nephrectomy samples from young/middle-aged reference patients, elderly patients, and individuals with diabetes or hypertension, the two most common underlying causes of CKD (Fig. 7a). CGRP levels were significantly reduced in kidneys from elderly and diabetic patients compared with those from reference patients, whereas levels in samples from hypertensive patients showed no significant difference (Fig. 7b).

We then modeled these conditions in mice using streptozotocin/high‑fat‑diet (HFD)‑induced diabetes and natural aging (Fig. 7c, e). Consistent with the human data, renal CGRP was lower at baseline and showed a blunted increase after I/R in both diabetic and aged mice compared with their respective controls (Fig. 7d, f). Two‑way ANOVA revealed significant main effects of diabetes and aging, as well as a significant main effect of I/R injury, in each of the two models, with significant interactions between diabetes or age and I/R (diabetes: *P* = 0.0172; aging: *P* = 0.002), indicating that both diabetes and aging impair the neuropeptide response to ischemic injury.

Given that aging and diabetes heighten susceptibility to I/R, we next tested whether CGRP supplementation mitigates this risk. In diabetic mice subjected to 25 min of bilateral I/R, CGRP treatment significantly reduced renal pro-inflammatory cytokines (TNF, IL-1β) and chemokines (CXCL2, CCL3) on day 2 (ELISA) and reduced the infiltration of neutrophils, Mo/Mφ, and classical monocytes (flow cytometry) (Fig. 7g, h; Supplementary information Fig. S9a–c). Consistent with these findings, CGRP improved both acute and chronic outcomes, as evidenced by functional and histopathological assessments of days 2 and 28 after I/R (Fig. 7i–l; Supplementary information Fig. S9d).

CGRP conferred a similar benefit in aged mice subjected to 30 min of bilateral I/R: treatment reduced levels of TNF, IL-1β, CXCL2, and CCL3 on day 2, durably curtailed the infiltration of neutrophils, Mo/Mφ, and classical monocytes, and ameliorated acute (day 2) and chronic (day 28) kidney injury as determined by functional and histological assessments (Fig. 7m–r; Supplementary information Fig. S9e–h).

Collectively, these findings suggest that impaired CGRP-mediated nociceptor signaling contributes to the heightened vulnerability of aged and diabetic kidneys to I/R-induced AKI and subsequent CKD progression and that restoring this pathway exerts a potent protective effect.

## Discussion

Kidney injury is a major clinical challenge, characterized by a high incidence, severe outcomes, and a lack of targeted therapies beyond supportive care.^1,4^ Immune mechanisms underlie AKI and its transition to CKD. Although sterile kidney injury is increasingly regarded as an inflammatory process, and myeloid immune cells have emerged as promising therapeutic targets, effective strategies remain elusive.^8,42^ Although nociceptors regulate immune function in diverse pathologies,^43^ their contribution to renal biology has been unclear.^44^ Here, we reveal that the kidney is densely innervated by nociceptive neurons and that renal I/R is associated with altered nociceptor morphology and increased renal CGRP abundance. This neuropeptide signals via the CALCRL–RAMP1 receptor on monocytes/macrophages to suppress pro-inflammatory mediators, thereby reducing renal inflammation and tubular damage. Collectively, our findings define the kidney-specific anatomical, functional, and myeloid signaling basis of this neuroimmune framework during I/R injury.

Renal innervation comprises efferent sympathetic fibers projecting to the kidney and afferent sensory fibers projecting to the spinal cord.^24,44^ Efferent sympathetic nerves are implicated in various renal diseases. For instance, sympathetic hyperactivity contributes to hypertension and has been targeted therapeutically.^45,46^ By contrast, primary afferents have been viewed as largely confined to the pelvic wall, the principal mechanosensory site, and are sensitive to chemical stimuli such as intrapelvic or intra-arterial adenosine, bradykinin, SP, PGE₂, and hypertonic saline.^44,47^ To date, preclinical studies have not provided definitive evidence for dedicated nociceptive afferents in the kidney or for sensory innervation of the renal parenchyma.^24,44^ Using SHANEL clearing and light-sheet microscopy, we revealed a dense parenchymal network of TRPV1^+^ nociceptive fibers extending beyond the collecting system. Renal I/R injury was associated with altered nociceptor terminal morphology and increased renal CGRP abundance, consistent with injury-associated changes in nociceptor activity and CGRP availability. Given the established neuroimmune roles of nociceptors in injury and repair, these findings support a potential contribution of sensory neuronal signaling to renal pathology.

Renal ischemia is the most common and central pathogenic mechanism underlying AKI, serving as a major driver in both clinical and experimental contexts.^48,49^ Accordingly, we used the bilateral renal I/R model to investigate nociceptor function and the underlying mechanism. Functionally, nociceptor ablation, whether systemic, DRG-targeted, or kidney-localized, exacerbated renal dysfunction, acute tubular injury, and subsequent fibrosis after I/R. This aligns with the anti-inflammatory actions of nociceptors reported in other contexts: in lung and joint inflammation, during infection when CGRP limits macrophage activation and neutrophil influx,^15,16,22,31^ and during tissue repair in skin and muscle.^23^ Consistent with this paradigm, nociceptor loss increased renal myeloid infiltration, whereas CGRP supplementation reduced both injury and myeloid recruitment. Our study extends this established principle to the kidney by defining the renal nociceptor network, its DRG origin, and its role in AKI-to-CKD progression.

By combining retrograde and anterograde tracing, we showed that each kidney receives robust, spatially segregated nociceptive input predominantly from ipsilateral T10–L2 DRGs. Selective manipulation of these segments provided complementary loss- and gain-of-function evidence: RTX-mediated ablation in bilateral T10–L2 DRGs aggravated I/R injury, whereas chemogenetic activation of T10–L2 TRPV1^+^ neurons was protective. In principle, sensory afferents from visceral organs can ascend to engage brain and cortical circuits.^35^ Although mechanosensitive and chemosensitive renal afferents have been implicated in systemic sequelae such as hypertension and cardiorenal dysfunction,^44,45,50^ the upstream injury-derived signals that activate renal nociceptors and trigger CGRP release remain to be defined. Future studies should map these pathways, establish their physiological relevance, determine how they interface with sympathetic outflow, and assess whether sensory and sympathetic pathways act synergistically to drive renal pathogenesis.

At the mechanistic level, CGRP emerged as the key mediator: it was the only neuropeptide among those examined that was abundantly expressed at baseline and further increased after I/R. Genetic loss of *Calca* (which encodes the αCGRP precursor) worsened injury, whereas exogenous CGRP improved both acute and chronic outcomes and rescued the phenotype of sensory denervation. Crucially, myeloid-restricted deletion of *Calcrl* (*Lyz2*^*ΔCalcrl*^) phenocopied sensory ablation, augmenting Mo/Mφ and neutrophil infiltration and worsening renal dysfunction and histological damage, and rendered mice unresponsive to exogenous CGRP. Reducing renal Mo/Mφ abundance abrogated the exaggerated inflammation and injury in *Lyz2*^*ΔCalcrl*^ mice, supporting Mo/Mφ as major cellular effectors of CGRP-mediated protection.

These findings are consistent with a broader principle in which nociceptors shape innate immunity in a context-dependent manner. During infection, nociceptors can restrain host defense, for example, by limiting neutrophil and γδ T cell responses in the lung^36^ and suppressing macrophage chemokines and neutrophil recruitment in meningitis.^31^ In sterile injury and repair, nociceptor signals promote resolution: CGRP–RAMP1 signaling reprograms myeloid cells toward repair in skin and muscle,^22^ and TAFA4 drives macrophage IL-10 production in UV-injured skin.^23^ In renal I/R, a paradigm of sterile, innate immune-driven inflammation,^42^ nociceptor-derived CGRP attenuated maladaptive myeloid responses, normalized leukocyte infiltration, and limited tubular damage. An open question is the relative contribution of specific phagocyte subsets — resident macrophages, circulating monocytes, and monocyte-derived macrophages — to this protective neuroimmune pathway.

Downstream, CALCRL–RAMP1 is Gs-coupled and elevates cAMP to activate PKA.^34,37,43^ By integrating RNA-seq of sorted renal Mo/Mφ with in vivo and in vitro perturbations, we showed that loss of CGRP signaling in myeloid cells upregulates neutrophil-attracting chemokines (CXCL1/2/12), monocyte chemoattractants (CCL2/3/4/5/7), and pro-inflammatory cytokines (TNF-α, IL-1β). In BMDMs, CGRP suppressed LPS-induced inflammatory transcripts, but this suppressive effect was absent in *Lyz2*^*ΔCalcrl*^ cells and was restored by forskolin, indicating that activation of the cAMP-PKA pathway is sufficient to induce the anti-inflammatory program even without CGRP receptors. In vivo, forskolin normalized cytokine/chemokine expression, reduced infiltration of classical monocytes and neutrophils, and ameliorated kidney injury after I/R in *Lyz2*^*ΔCalcrl*^ mice, placing cAMP-PKA downstream of CALCRL–RAMP1 in this axis.

Finally, we extended these insights to clinically relevant settings. Diabetes is the leading cause of CKD,^51–53^ and the prevalence of CKD rises steeply with age. Analysis of nephrectomy specimens demonstrated significantly lower renal CGRP levels in diabetic and aged kidneys, revealing a previously underappreciated reduction in renal CGRP. Mouse models of aging and diabetes recapitulated the reduced baseline CGRP levels and blunted I/R‑induced rise. Two‑way ANOVA confirmed significant interactions between diabetes or age and injury in both models, suggesting that diabetes and aging impair the neuropeptide response to ischemic stress, which may contribute to the heightened susceptibility of these kidneys to acute injury and subsequent fibrosis. CGRP supplementation reduced levels of inflammatory mediators, reduced myeloid and neutrophil infiltration, and improved both acute and chronic outcomes in these vulnerable models, and these protective effects were more pronounced than those in young healthy mice. Elderly and diabetic patients are at high risk for AKI and CKD, in part because aging diminishes renal reserve^41^ and diabetes drives metabolic and inflammatory injury.^51^ Our findings suggest that impaired CGRP-mediated nociceptor signaling is an underappreciated contributor to the heightened susceptibility of aged and diabetic kidneys to I/R-induced AKI and its progression to CKD and that restoring this pathway holds translational promise for these vulnerable populations.

All formal mechanistic and longitudinal in vivo experiments were conducted in female mice; therefore, the conclusions are restricted to females. Whether the magnitude, mechanism, or therapeutic responsiveness of the nociceptor-CGRP pathway differs in males remains to be determined. We did not experimentally address the upstream signals that drive CGRP release from DRG neurons; both aspects warrant future investigation. Our anatomical mapping does not resolve the central projections of renal nociceptors or fully delineate interactions with sympathetic outflow. PRV-based tracing, although informative, has potential limitations related to trans-synaptic spread, and TRPV1-lineage tools label a subset of nociceptors. Future studies that integrate activity-resolved imaging, single-cell multi-omics of renal afferents and myeloid subsets, and human functional validation will refine therapeutic targeting. Nonetheless, our findings identify a kidney-protective nociceptor-myeloid circuit in which DRG-derived CGRP, acting via CALCRL–RAMP1 and cAMP-PKA, restrains maladaptive inflammation. Targeting this nociceptive neuroimmune axis represents a plausible strategy for treating AKI and halting its progression to CKD, particularly in high-risk diabetic and elderly populations.

## Materials and methods

### Mice

*Trpv1-Cre* (C57BL/6-*Trpv1*^em1(Myc-IRES-Cre)Smoc^, NM-KI-200139) mice, *iEGFP* (*Rosa26-CAG-LSL-Luc-2A-EGFP*, NM-KI-190095) mice, and *Lyz2-Cre* (B6N.129P2-*Lyz2*^*tm1(cre)Ifo*^*/J*^+^^*/–*^, Jax 018956) mice were generated on a C57BL/6 background and provided by Shanghai Model Organisms Center. *Calcrl*^*fl/fl*^ (C57BL/6J-*Calcrl*^*em1flox*^/Cya, S-CKO-11816), *Calca*^*−/−*^ (C57BL/6N-*Calca*^*em1*^/Cya, S-KO-01299), *iDTR* (*Rosa26-LSL-DTR*, I001040), and *itdTomato* (*Rosa26-LSL-tdTomato*, C001742) mice were generated on a C57BL/6 background and provided by Cyagen Biosciences (Suzhou, China). Wild-type C57BL/6 mice were purchased from Beijing Vital River Laboratory Animal Technology. *Trpv1-Cre* mice were crossed with *iEGFP* mice to generate *Trpv1-Cre*^*+*^*iEGFP*^*+*^ mice. *Trpv1-Cre* heterozygous mice were bred with *iDTR* mice to generate *Trpv1-Cre*^*+*^*iDTR*^*+*^ mice for subsequent nociceptor ablation and control littermates (*Trpv1-Cre*^*−*^*iDTR*^*+*^). For conditional knockout experiments, *Lyz2-Cre* mice were crossed with *Calcrl*^*fl/fl*^ mice to generate *Lyz2-Cre*^+^^*/–*^*Calcrl*^*fl/fl*^ mice (hereafter *Lyz2*^*ΔCalcrl*^) with myeloid-specific deletion of *Calcrl*; Cre-negative *Calcrl*^*fl/fl*^ littermates were used as controls. Mice were maintained under specific-pathogen-free (SPF) conditions with controlled temperature (21–23 °C), humidity (30%–60%), and a 12-h light/dark cycle. All formal mechanistic and longitudinal in vivo experiments were performed in female mice. Female mice were selected because bilateral I/R produced reproducible AKI-to-CKD progression with relatively low mortality, whereas exploratory experiments in male mice showed high mortality at injury-inducing ischemic durations (Supplementary information Fig. S2d–f). Accordingly, conclusions from the formal mouse experiments apply to females and should not be generalized to males. Unless otherwise specified, mice subjected to renal I/R surgery were 8–10 weeks old. In the aging and diabetes experiments, aged mice were 20 months old, and diabetic mice underwent I/R surgery at 15–17 weeks of age. All animal procedures were approved by the Institutional Animal Care and Use Committee of the Chinese People’s Liberation Army General Hospital.

### Clinical specimens

Human kidney tissue specimens were consecutively collected from the Urological Surgery Kidney Biobank of the Chinese PLA General Hospital over a one-year period (January–December 2019). The collection and use of human samples were approved by the Ethics Committee of the Chinese PLA General Hospital (approval no. S2013-065-01), and written informed consent was obtained from all participants before inclusion. The use of these archived specimens in the present study was reviewed and exempted from additional ethical review by the Ethics Committee of the Chinese PLA General Hospital (approval no. S2023-428-01). All specimens were obtained during nephrectomy for renal tumors from macroscopically normal cortical regions located at least 0.5 cm from the tumor margin.

The study cohort (*N* = 48) consisted of four groups (*n* = 12 per group):

1. young/middle-aged reference patients (< 60 years);
2. patients with diabetes who were < 60 years old and had a > 5-year history of diabetes or clinically diagnosed diabetic kidney disease according to KDIGO guidelines^54^;
3. hypertensive patients (< 60 years, with > 5-year history of stage 2 or higher hypertension or clinically diagnosed hypertensive nephropathy); and
4. elderly patients (≥ 65 years).

Immediately after surgical resection, renal cortical tissues were snap-frozen in liquid nitrogen and stored at −80 °C until analysis. For CGRP quantification, frozen kidney samples were homogenized in ice-cold PBS containing protease inhibitors, and supernatants were collected after centrifugation. CGRP levels were measured using a commercial ELISA kit (No. 589101; Cayman Chemical) according to the manufacturer’s instructions.

### Surgical model of renal ischemic injury

Renal I/R injury was induced by bilateral renal pedicle clamping. The duration of ischemia was adjusted to modulate injury severity: 25 min for diabetic mice, 30 min for aged mice, and 35 min for all other experimental groups. For the exploratory male experiment shown in Supplementary information Fig. S2d–f, male mice underwent 30 min of bilateral renal I/R. Mice were anesthetized and maintained on a heating platform at 37 °C throughout the procedure. Through a dorsal incision, both renal pedicles were clamped with non-traumatic microvascular clamps until a uniform darkening of the kidneys was observed. After the indicated ischemia duration, clamps were released to allow reperfusion, which was confirmed by the return of uniform pink coloration. Mice were excluded if the kidneys failed to darken within 5–10 min of clamping or did not reperfuse promptly after clamp removal. Fluid loss was compensated for by intraperitoneal injection of 500 µL of 0.9% NaCl. Animals were euthanized on day 2 or 28 after surgery to assess AKI and CKD, respectively. Sham-operated controls underwent identical procedures except for clamping.

### Kidney injury evaluation

For functional assessment, blood samples were collected and centrifuged at 8000 rpm for 5 min at 4 °C to obtain serum. Serum creatinine and urea concentrations were measured at the Department of Laboratory Medicine, PLA General Hospital, using a Roche Cobas c701 automated chemistry analyzer. The serum samples were diluted 1:1 with distilled water before measurement.

For morphological evaluation, kidneys were fixed in 10% neutral-buffered formalin, embedded in paraffin, and sectioned for PAS and Masson’s trichrome staining. Histopathological evaluation was performed by two renal pathologists blinded to experimental conditions. The acute tubular necrosis (ATN) score was determined by quantifying the percentage of tubules exhibiting necrosis, brush border loss, luminal dilation, or cast formation in PAS-stained sections: 0, none; 1, 1%–10%; 2, 11%–25%; 3, 26%–45%; 4, 46%–75%; and 5, 76%–100%. Interstitial fibrosis was quantified on Masson’s trichrome-stained kidney sections. For each section, five non-overlapping fields (400×) from the corticomedullary junction and five non-overlapping fields from the cortex were selected (a total of 10 fields per section), excluding large vessels and the renal pelvis. After threshold adjustment using Image-Pro Plus software (Media Cybernetics, Rockville, MD, USA), the average percentage of blue-stained area was calculated. All thresholds were set by an investigator blinded to group allocation and remained constant across all sections. Collagen I immunostaining was quantified in the same manner, by measuring the percentage of brown-stained area using ImageJ as previously described.^55^

### Kidney clearing with SHANEL

Whole-mount immunolabeling of the kidney was performed using an optimized SHANEL protocol.^30,56,57^ In brief, anesthetized mice were transcardially perfused with PBS followed by 4% paraformaldehyde (PFA). The kidneys were then dissected and post-fixed in 4% PFA at 4 °C for 12 h. The fixed tissues were subsequently permeabilized and decolorized in a CHAPS/NMDEA solution. A series of dehydration and rehydration steps was carried out by sequentially rinsing the samples in 50%, 70%, and 100% ethanol, a DCM/MeOH solution, 100%, 70%, and 50% ethanol, and finally deionized water. Following delipidation, the samples were treated with a 0.5 M acetic acid solution and a guanidine solution. After blocking, the samples were incubated with rabbit anti-CGRP (1:400; Cat# C8198; Sigma) or rabbit anti-tyrosine hydroxylase (1:400; Cat# AB152; Merck) primary antibodies for 3 days at 37 °C. After washing, the samples were immunolabeled with the corresponding Alexa Fluor-conjugated secondary antibodies (1:400; Cat# A11035; Thermo Fisher Scientific) for another 3 days at 37 °C. Following extensive washes, the samples were dehydrated through a graded ethanol series (50%, 70%, 100%, 100%) and delipidated with pure DCM. Finally, the immunolabeled kidneys were rendered transparent by refractive index (RI) matching through incubation in BABB solution.

### Kidney clearing with advanced CUBIC

Whole-mount immunolabeling of selected kidney samples was performed using an advanced CUBIC protocol.^57,58^ Following intracardiac perfusion and kidney collection as described above, kidneys were sectioned into ~2-mm-thick slices using a razor blade. Tissue slices were cleared by incubation in half-diluted CUBIC-L solution overnight at 37 °C, followed by two incubations in full-strength CUBIC-L solution for 1 day each at 37 °C. After clearing, the samples were blocked and incubated with chicken anti-GFP (1:200; Cat# GFP-1020; Aves Labs) and rabbit anti-CGRP (1:400; Cat# C8198; Sigma) primary antibodies for 2 days at 37 °C. Following thorough washes, tissues were incubated with an Alexa Fluor 647-conjugated goat anti-chicken IgY (H + L) secondary antibody (1:400; Cat# A-21449; Thermo Fisher Scientific) and an Alexa Fluor 546-conjugated goat anti-rabbit IgG (H + L) cross-adsorbed secondary antibody (1:400; Cat# A-11035; Thermo Fisher Scientific) for 1 day at 37 °C. After a final wash, the immunolabeled samples were rendered optically transparent by RI matching in CUBIC-R+(M) solution (two successive incubations). Cleared kidney slices were imaged using a Zeiss LSM 900 confocal microscope or light‑sheet fluorescence microscopy, as described in detail in the following section.

### Light-sheet fluorescence microscopy and 3D reconstruction

Optically cleared whole-mount kidneys were immersed in an imaging chamber filled with BABB or CUBIC-R+(M) solution. Imaging was performed using one of the following light-sheet fluorescence microscopy systems: a Zeiss Lightsheet Z.1 equipped with dual-sided 5×/0.1 NA illumination and 5×/0.16 NA detection optics; a Zeiss Lightsheet 7 equipped with dual-sided 10×/0.2 NA illumination and 20×/1.0 NA detection optics; or a Luxendo MuVi SPIM-CS (Bruker) equipped with dual-sided 10×/0.3 NA illumination and 10×/0.5 NA detection optics. Image stacks were acquired using a continuous light-sheet scanning mode.

Samples were sequentially scanned from both illumination directions, generating paired single-sided image sets, which were fused using the Dual Side Fusion function in Zeiss ZEN 3.2 (Blue edition). 3D reconstruction, volume rendering, and quantitative image analysis were performed in Imaris v9.9.0 (Bitplane). The density of nociceptive or sympathetic nerve fibers was quantified as the ratio of labeled nerve fiber volume to total kidney volume, as determined by isosurface masking.

### Retrograde and anterograde neuronal tracing

Retrograde neuronal tracing was performed using the Bartha strain of pseudorabies virus (PRV-Ba), an attenuated live vaccine strain commonly used as a trans-synaptic tracer. Adult C57BL/6 mice were anesthetized with isoflurane, and the left kidney was exposed through a left subcostal incision. Using a Nanoliter 2020 injector (World Precision Instruments), a total of 3 μL of PRV-CMV-GFP viral stock (5 × 10^9^ viral genomes/mL, Cat# BC-PRV-531; Brain Case) was slowly injected into multiple sites in the kidney parenchyma to achieve even viral distribution. To prevent leakage into the peritoneal cavity, each injection site was immediately covered with a sterile cotton swab. The muscle and skin layers were then closed with standard sutures. Two days after injection, bilateral thoracic/lumbar DRGs and NGs were collected, processed for whole-mount TRPV1 immunostaining, and optically cleared using the advanced CUBIC method.

For anterograde labeling of nociceptive projections, AAV9-Trpv1-SV40-NLS-Cre (5.0 × 10^12^ viral genomes/mL, Cat# BC-0306; Brain Case) was microinjected into the left or right T10–L2 DRGs of itdTomato^+^ mice. Intra-ganglionic injection was performed as described previously.^59^ Four weeks after injection, ipsilateral kidneys were processed for anti-CGRP immunostaining and optical clearing using the advanced CUBIC protocol.

### Nociceptor ablation and activation

#### Systemic nociceptor ablation

Systemic ablation of nociceptors was achieved using either a genetic or a chemical approach. For genetic ablation, *Trpv1-Cre*^+^*iDTR*^+^ mice and littermate *Trpv1-Cre*^−^*iDTR*^+^ controls received intraperitoneal injections of DT (200 ng in 100 μL PBS; Cat# D0564; Sigma) according to a cyclic regimen (5 days on, 2 days off) for 21 days, as described previously.^57,60^ Mice were allowed a one-week recovery period before subsequent experiments.

For chemical ablation, 4-week-old female C57BL/6 mice received escalating subcutaneous doses of RTX (30 μg/kg, 70 μg/kg, and 100 μg/kg; Cat# R8756; Sigma) or vehicle (PBS containing 1.2% DMSO and 0.06% Tween-80) on three consecutive days under light isoflurane anesthesia. Experiments were performed four weeks post-injection.

#### Kidney-localized nociceptor ablation

Localized renal sensory denervation was induced by topical application of high-concentration capsaicin (33 mM, Cat# HY-10448; MedChemExpress). Following dorsal surgical exposure of the renal artery and peri-arterial innervation under a stereomicroscope, the target area was gently grasped five times (10 s each) with micro-forceps wrapped in cotton pre-soaked with capsaicin or vehicle solution. The surgical field was then thoroughly rinsed with sterile saline. Mice were allowed to recover for two weeks before further procedures.

#### Vagal-targeted ablation

For vagal-specific ablation, the vagal ganglia of anesthetized *Trpv1-Cre*^+^*iDTR*^+^ or *Trpv1-Cre*^−^*iDTR*^+^ mice were exposed through a 1-cm ventral midline cervical incision. Bilateral intra-ganglionic injections of DT (20 ng DT in 120 nL PBS containing 0.05% Fast Green; Cat# D0564; Sigma) were administered using a Nanoliter 2020 Injector (World Precision Instruments).^61^ Mice were allowed at least two weeks to recover before renal I/R surgery.

#### DRG-targeted ablation and activation

To ablate TRPV1^+^ nociceptors innervating the kidneys, bilateral T10–L2 DRGs of 6-week-old C57BL/6 mice were injected intra-ganglionically with RTX (3 ng in 200 nL of vehicle: 0.25% DMSO, 0.02% Tween-80, and 0.05% ascorbic acid in PBS) or vehicle alone.^59^ Mice were allowed to recover for four weeks before experimentation.

For chemogenetic activation, *Trpv1-Cre*^+^ mice received bilateral T10–L2 DRG microinjections of a Cre-dependent excitatory DREADD vector (AAV9-CAG-DIO-hM3D(G_q_)-mCherry; 1.3 × 10¹³ viral genomes/mL, 200 nL per ganglion; Cat# BC-0395; Brain Case). Mice were allowed four weeks for recovery and transgene expression before undergoing renal I/R surgery. CNO (3 mg/kg, intraperitoneal administration; Cat# C0832; Sigma) or PBS vehicle was administered daily, starting two days before I/R induction and continuing for 14 days after surgery.

### Whole-mount ganglion staining, imaging, and analysis

#### Tissue collection, clearing, and immunostaining

Retrogradely traced mice were perfused intracardially with PBS followed by 4% PFA on day 2 after injection or at the indicated time points. Thoracic and lumbar DRGs and bilateral NGs were collected and post-fixed overnight in 4% PFA at 4 °C. Fixed ganglia were cleared by incubation in CUBIC-L solution at 37 °C for 6 h, permeabilized in PTxwH buffer (PBS containing 0.5% Triton X-100, 0.05% Tween-20, and 4 mg/mL heparin) for 4 h, and blocked in 5% normal goat serum. For retrograde tracing combined with TRPV1 labeling and for assessing nociceptor ablation efficiency, tissues were incubated with rabbit anti-TRPV1 (1:400, Cat# ACC-030; Alomone Labs) for 48 h at 4 °C. After washing, samples were incubated with an Alexa Fluor 647-conjugated goat anti-rabbit secondary antibody (1:400) for 24 h at 4 °C. For dual-color quantification of CGRP alongside TRPV1, tissues were co-incubated with a mixture of two primary antibodies: rabbit anti-TRPV1 (1:400) and human anti-CGRP (1:400, Cat# B587301; Bio-Compare). The tissues were then incubated with a mixture of Alexa Fluor 647-conjugated goat anti-rabbit (1:400) and Alexa Fluor 546-conjugated goat anti-human (1:400) secondary antibodies for 24 h at 4 °C.

#### Imaging

After extensive washes, samples were rendered optically transparent by RI matching in CUBIC-R+(M) solution (two incubations). Cleared ganglia were imaged using a Zeiss LSM 900 confocal microscope equipped with a 10×/0.45 NA objective. Each ganglion was imaged using sequential z-stacks encompassing its entire volume.

#### Quantitative analysis

For retrograde tracing experiments, all z-stacks from each ganglion were analyzed to quantify PRV^+^TRPV1^+^ neurons. To assess nociceptor ablation efficiency, TRPV1^+^ neurons were counted in three *z*-stacks per DRG, spaced ≥ 20 μm apart across the entire ganglion. Counts were obtained from a central field of view (400 μm × 400 μm) per *z*-stack, and data were expressed as the average number of TRPV1^+^ neurons per field. To quantify CGRP expression, the proportion of CGRP^+^ neurons among TRPV1^+^ neurons was calculated from three representative optical sections per mouse. All analyses were performed by investigators blinded to genotype and treatment.

### Measurement of renal neuropeptides by ELISA

Renal concentrations of CGRP, VIP, SP, and NE were quantified in kidney homogenates. Tissue samples were thawed, homogenized, and centrifuged at 8000× *g* for 5 min at 4 °C. Supernatants were analyzed using commercial ELISA kits according to the manufacturers’ instructions: CGRP, VIP, SP, and NE EIA Kits (No. 589001, CSB-E08356m, No. 583751, KA1877-1; Cayman Chemical). Analyte concentrations were normalized to kidney weight and expressed as mass per gram of tissue.

### Measurement of renal inflammatory mediators by ELISA

Renal cytokine and chemokine levels were determined in kidney homogenate supernatants prepared as described above. TNF, IL-1β, CXCL2, and CCL3 concentrations were measured using commercial ELISA kits (TNF, E-EL-M3063-96T, Elabscience; IL-1β, E-EL-M0037c-96T, Neobioscience; CXCL2, E-EL-M0019-96T, Neobioscience; CCL3, E-EL-M0007-96T, Neobioscience) following the manufacturers’ protocols. Results were normalized to kidney weight and expressed as mass concentrations.

### Renal flow cytometry

Anesthetized mice were perfused intracardially with PBS, and kidneys were excised, minced, and digested for 40 min at 37 °C in HBSS (with Ca²^+^/Mg²^+^) containing collagenase IV (0.75 mg/mL) and DNase I (20 μg/mL). The digestion was quenched with RPMI-1640 medium supplemented with 10% fetal bovine serum (FBS) and 1% penicillin/streptomycin, filtered through a 70-μm strainer, and treated with RBC Lysis Buffer (Cat# 00-4300-54; Thermo Fisher Scientific). After cell counting, single-cell suspensions were blocked with anti-mouse CD16/32 Fc Block (clone 93; Cat# 14-0161-86; Thermo Fisher Scientific) and stained for 30 min on ice in flow buffer (2% FBS and 2 mM EDTA in PBS) with the following reagents: Fixable Viability Dye eFluor 780 (1:1000; Cat# 65-0865-14; Thermo Fisher Scientific), PerCP anti-mouse CD45 (1:200; clone 30-F11; Cat# 103130; BioLegend), BV421 anti-mouse/human CD11b (1:200; clone M1/70; Cat# 101236; BioLegend), PE/Cyanine7 anti-mouse Ly6C (1:200; clone HK1.4; Cat# 128018; BioLegend), APC/Cyanine7 anti-mouse CD11c (1:200; clone N418; Cat# 117324; BioLegend), APC anti-mouse F4/80 (1:200; clone BM8; Cat# 123116; BioLegend), FITC anti-mouse Ly6G (1:200; clone 1A8; Cat# 127606; BioLegend), BV605 anti-mouse CD3 (1:200; clone 17A2; Cat# 100237; BioLegend), FITC anti-mouse CD19 (1:200; clone MB19-1; Cat# 11-0191-82; Thermo Fisher Scientific), APC anti-mouse XCR1 (1:200; clone ZET; Cat# 148205; BioLegend), and PE/Cyanine7 anti-mouse CD64 (1:200; clone X54-5/7.1; Cat# 139313; BioLegend). Dead cells were excluded using Fixable Viability Dye eFluor 780. Separate antibody panels were used for the analysis of different immune-cell subsets. Antibodies that shared the same fluorophore were used in mutually exclusive panels and were not included together in the same staining tube. Data were acquired on a BD LSRFortessa or FACSAria III flow cytometer using FACSDiva v8.0.2 and analyzed with FlowJo v10.0.7 (TreeStar). Gating strategies are shown in Supplementary information, Fig. S6a.

### Isolation of renal monocytes and macrophages

Renal monocytes/macrophages (Mo/Mφ) were isolated by a two-step purification procedure. Single-cell suspensions were prepared as described above, resuspended in wash buffer (BioLegend), and passed through a 40-μm strainer. Myeloid cells were initially enriched from the single-cell suspension using MACS with CD11b MicroBeads UltraPure (Cat# 130-097-142; Miltenyi Biotec) and MS columns, following the manufacturer’s instructions. The MACS-enriched CD11b^+^ cells were immediately subjected to FACS. Single-cell suspensions were blocked with anti-CD16/32 (25 μg/mL, 10 min) and stained for 30 min on ice in flow buffer with the following reagents: BV421 anti-mouse/human CD11b (1:200; clone M1/70), APC/Cyanine7 anti-mouse CD11c (1:200; clone N418), FITC anti-mouse Ly6G (1:200; clone 1A8), and Fixable Viability Dye eFluor 780. After washing, the target renal Mo/Mφ population (CD11b^hi^ Ly6G^−^ CD11cˡᵒ) was sorted on a BD FACSAria III flow cytometer (85-μm nozzle, 4 °C) for subsequent RNA-seq or qPCR analysis.

### RNA-seq of renal monocytes and macrophages

Renal monocytes and macrophages (Mo/Mφ) were isolated from *Lyz2*^*ΔCalcrl*^ mice and *Calcrl*^*fl/fl*^ littermate controls two days after bilateral renal I/R injury, using the two-step purification procedure described above. Total RNA was extracted from the sorted cells using the RNeasy Plus Micro Kit (Cat# 74034; Qiagen) according to the manufacturer’s protocol. RNA integrity and concentration were assessed using an Agilent Bioanalyzer 2100 system. Library preparation and sequencing were performed by Annoroad Gene Technology (Beijing, China), Libraries were prepared using the Smart-Seq2 protocol and sequenced on an Illumina NovaSeq SP platform, generating paired-end reads (150 bp).

### Analysis of DEGs

Raw sequencing reads were quality-checked and trimmed before alignment to the *Mus musculus* reference genome (GRCm39) using STAR (v2.7). Read counts were quantified with featureCounts, and normalization and differential expression analysis were performed using the R package DESeq2 (v1.38). Genes with an adjusted *P* value < 0.05 (Benjamini–Hochberg correction) were considered differentially expressed. Volcano plots and heatmaps were generated using the EnhancedVolcano and pheatmap R packages, respectively.

### Pathway enrichment analysis

Functional enrichment analyses were performed using clusterProfiler (v4.6.2). For gene annotation, the org.Mm.eg.db database was used to assign Entrez Gene IDs. DEGs were analyzed using the Kyoto Encyclopedia of Genes and Genomes (KEGG) and Gene Ontology (GO) databases. Significantly enriched pathways were defined as those with a false discovery rate (FDR)-adjusted *P* value < 0.25, according to the Benjamini–Hochberg method. Non-redundant GO terms and KEGG pathways were selected for visualization in bubble plots and bar graphs.

### Isolation and culture of BMDMs and in vitro treatment

BMDMs were generated from bone marrow collected from the femurs and tibias of *Lyz2*^*ΔCalcrl*^ mice or *Calcrl*^*fl/fl*^ mice. In brief, bone marrow cells were flushed out with cold Dulbecco’s modified Eagle’s medium (DMEM; Gibco) supplemented with 10% heat-inactivated FBS (Gibco), 1% penicillin–streptomycin (Gibco), and 20 ng/mL recombinant murine macrophage colony-stimulating factor (M-CSF; PeproTech). Cells were cultured in non-tissue-culture-treated Petri dishes at 37 °C in a humidified 5% CO₂ incubator for 7 days, with medium replacement on day 4. On day 7, fully differentiated macrophages were harvested and seeded into 12-well plates (1 × 10⁶ cells per well) for stimulation assays. For inflammatory activation, BMDMs were stimulated with LPS (100 ng/mL; Sigma) for 4 h in serum-free DMEM. To evaluate the immunomodulatory effects of CGRP or cAMP-PKA activation, cells were treated with CGRP (100 nM; Cat# RP11095; GenScript) or forskolin (10 μM; Cat# HY-15371; MedChemExpress), together with LPS. Vehicle-treated cells served as controls. After stimulation, cells were washed twice with cold PBS. Total RNA was isolated using the RNeasy Mini Kit (Qiagen) for qPCR analysis of pro-inflammatory genes (*Tnf*, *Il1b*, *Cxcl2*, and *Ccl3*).

### Drug treatments

To pharmacologically deplete kidney monocytes/macrophages, mice were fed an AIN‑76 A diet containing PLX5622 (MCE) (1.2 g PLX5622 per kg of diet, formulated by SYSE Bio or Research Diets) ad libitum.^62^ Control mice were fed a standard AIN‑76 A diet (control diet, CD) (SYSE Bio or Research Diets) ad libitum. In some experiments, mice were injected intraperitoneally with 500 µg/kg CGRP (GenScript). In other experiments, mice were injected intraperitoneally daily with 10 mg/kg forskolin (MCE) in 200 μL of vehicle.

### Diabetic model and natural aging model

A murine model of diabetes was established by combining an HFD with low-dose streptozotocin (STZ) administration. In brief, 6–8-week-old C57BL/6 mice were fed either an HFD or standard chow for 4 weeks, followed by daily intraperitoneal injections of STZ (40 mg/kg/day; Sigma) or citrate buffer vehicle for 5 consecutive days. Fasting blood glucose levels were measured using a glucometer (Roche) starting 1–2 weeks after the final STZ injection. Mice with fasting glucose levels ≥ 11.1 mM on two consecutive measurements were classified as diabetic. Diabetic mice were maintained on their respective diets for an additional 4 weeks before experimental use, which included quantification of renal neuropeptides or induction of renal I/R injury. Aging-related experiments were performed using naturally aged female C57BL/6 mice (20 months old) and young adult controls (8–10 weeks old).

### RNA extraction, reverse transcription, and qPCR

Total cellular RNA was extracted using the RNeasy Mini Kit (Cat# 74104; Qiagen) according to the manufacturer’s instructions. For small numbers of renal monocytes/macrophages (Mo/Mφ) purified by combined MACS and FACS, RNA was isolated using the RNeasy Plus Micro Kit (Qiagen). Complementary DNA was synthesized using HiScript III RT SuperMix (Vazyme) following the manufacturer’s protocol. qPCR was performed on a CFX96 Real-Time PCR Detection System (Bio-Rad) using Taq Pro Universal SYBR qPCR Master Mix (Vazyme) under the following cycling conditions: 95 °C for 30 s, followed by 40 cycles of 95 °C for 5 s and 60 °C for 30 s. *Gapdh* served as the internal reference gene, and relative mRNA expression levels were calculated using the 2^−^^ΔΔCt^ method. The following primer sequences (5′ to 3′) were used: *Gapdh* forward: CAGGAGAGTGTTTCCTCGTCC, *Gapdh* reverse: GATGGGCTTCCCGTTGATGA; *Tnf* forward: CAGGCGGTGCCTATGTCTC, *Tnf* reverse: CGATCACCCCGAAGTTCAGTAG; *Il1b* forward: CCAAAAGATGAAGGGCTGCT, *Il1b* reverse: TCATCAGGACAGCCCAGGTC; *Cxcl1* forward: CAATGAGCTGCGCTGTCAGT, *Cxcl1* reverse: TTGAGGTGAATCCCAGCCAT; *Cxcl2* forward: AAGTGTGAAGGTGAAGTCC, *Cxcl2* reverse: CGAGACTTACATGACTTCGG; *Cxcl10* forward: GCCGTCATTTTCTGCCTCA, *Cxcl10* reverse: CGTCCTTGCGAGAGGGATC; *Ccl2* forward: CCTGCTGCTACTCATTCACCA, *Ccl2* reverse: ATTCCTTCTTGGGGTCAGCA; *Ccl3* forward: TTCTCTGTACCATGACACTCTGC, *Ccl3* reverse: CGTGGAATCTTCCGGCTGTAG; *Ccl4* forward: TTCCTGCTGTTTCTCTTACACCT, *Ccl4* reverse: CTGTCTGCCTCTTTTGGTCAG; *Ccl5* forward: GTGCCCACGTCAAGGAGTAT, *Ccl5* reverse: CTCTGGGTTGGCACACACTT; *Ccl7* forward: GAGGATCTCTGCCACGCTTC, *Ccl7* reverse: ACACCGACTACTGGTGATCC.

### Quantification and statistical analysis

All statistical analyses and data visualization were performed using Prism software version 9.5.0 (GraphPad Software). Sample sizes (*n*) for each experiment are specified in the corresponding figure legends. For comparisons between two groups with normally distributed data, unpaired two-tailed *t*-tests were used. When variances between groups were unequal (as determined by an *F*-test), Welch’s correction was applied. For non-normally distributed data, statistical significance was assessed using the Mann–Whitney U test. For experiments involving more than two groups and one independent variable, one-way analysis of variance (ANOVA) followed by Dunnett’s or Tukey’s multiple comparisons test was used, as specified in the figure legends. For experiments involving two independent variables, two‑way ANOVA was used, followed by Šidák’s multiple comparisons test for selected pairs. Data are presented as the mean ± SEM, unless otherwise indicated. A *P* value < 0.05 was considered statistically significant.

## Supplementary information

Supplementary information, Video S1

Supplementary information, Video S2

Supplementary information, Fig. S1

Supplementary information, Fig. S2

Supplementary information, Fig. S3

Supplementary information, Fig. S4

Supplementary information, Fig. S5

Supplementary information, Fig. S6

Supplementary information, Fig. S7

Supplementary information, Fig. S8

Supplementary information, Fig. S9

## Data Availability

Bulk RNA-seq datasets generated and analyzed during this study have been deposited into the NCBI BioProject under accession number PRJNA1367940.

## References

[1] Ostermann, M. et al. Acute kidney injury. *Lancet* **405**, 241–256 (2025). 10.1016/S0140-6736(24)02385-7 39826969

[2] Hoste, E. A. J. et al. Global epidemiology and outcomes of acute kidney injury. *Nat. Rev. Nephrol.* **14**, 607–625 (2018). 10.1038/s41581-018-0052-0 30135570

[3] Riccardi, M., Pagnesi, M., Lombardi, C. M. & Metra, M. Severe acute kidney injury in the intensive care unit: step-to-step management. *Eur. Heart J. Acute Cardiovasc. Care* **14**, 618–630 (2025). 10.1093/ehjacc/zuaf084 40578821

[4] Zhang, T., Widdop, R. E. & Ricardo, S. D. Transition from acute kidney injury to chronic kidney disease: mechanisms, models, and biomarkers. *Am. J. Physiol. Ren. Physiol.* **327**, F788–F805 (2024). 10.1152/ajprenal.00184.2024 39298548

[5] Francis, A. et al. Chronic kidney disease and the global public health agenda: an international consensus. *Nat. Rev. Nephrol.* **20**, 473–485 (2024). 10.1038/s41581-024-00820-6 38570631

[6] Pickkers, P., Murray, P. T. & Ostermann, M. New drugs for acute kidney injury. *Intensive Care Med.* **48**, 1796–1798 (2022). 10.1007/s00134-022-06859-y PMC9705447 35999470

[7] Ostermann, M. & Liu, K. Pathophysiology of AKI. *Best Pract. Res. Clin. Anaesthesiol.* **31**, 305–314 (2017). 10.1016/j.bpa.2017.09.001 29248138

[8] Foresto-Neto, O., Menezes-Silva, L., Leite, J. A., Andrade-Silva, M. & Camara, N. O. S. Immunology of kidney disease. *Annu. Rev. Immunol.* **42**, 207–233 (2024). 10.1146/annurev-immunol-090122-045843 38211945

[9] Lazzeri, E. & Romagnani, P. Tubular cell polyploidy and AKI-to-CKD transition. *J. Am. Soc. Nephrol.* **36**, 751–754 (2025). 10.1681/ASN.0000000696 PMC12059084 40202799

[10] Levey, A. S. Defining AKD: The spectrum of AKI, AKD, and CKD. *Nephron* **146**, 302–305 (2022). 10.1159/000516647 34167119

[11] Lee, K., Jang, H. R. & Rabb, H. Lymphocytes and innate immune cells in acute kidney injury and repair. *Nat. Rev. Nephrol.* **20**, 789–805 (2024). 10.1038/s41581-024-00875-5 39095505

[12] Meng, X. M., Wang, L., Nikolic-Paterson, D. J. & Lan, H. Y. Innate immune cells in acute and chronic kidney disease. *Nat. Rev. Nephrol.* **21**, 464–482 (2025). 10.1038/s41581-025-00958-x 40263532

[13] Qu, L. & Jiao, B. The interplay between immune and metabolic pathways in kidney disease. *Cells* **12**, 1584 (2023). 10.3390/cells12121584 PMC10296595 37371054

[14] Rayego-Mateos, S. et al. Targeting inflammation to treat diabetic kidney disease: the road to 2030. *Kidney Int.* **103**, 282–296 (2023). 10.1016/j.kint.2022.10.030 36470394

[15] Sterner-Kock, A. et al. Neonatal capsaicin treatment increases the severity of ozone-induced lung injury. *Am. J. Respir. Crit. Care Med.* **153**, 436–443 (1996). 10.1164/ajrccm.153.1.8542155 8542155

[16] Strausbaugh, H. J. et al. Painful stimulation suppresses joint inflammation by inducing shedding of L-selectin from neutrophils. *Nat. Med.* **5**, 1057–1061 (1999). 10.1038/12497 10470085

[17] Hakim, S., Jain, A. & Woolf, C. J. Immune drivers of pain resolution and protection. *Nat. Immunol.* **25**, 2200–2208 (2024). 10.1038/s41590-024-02002-9 39528810

[18] Oshaghi, M., Kourosh-Arami, M. & Roozbehkia, M. Role of neurotransmitters in immune-mediated inflammatory disorders: a crosstalk between the nervous and immune systems. *Neurol. Sci.* **44**, 99–113 (2023). 10.1007/s10072-022-06413-0 36169755

[19] Crosson, T., Bretaud, N. & Ugolini, S. Role of specialized sensory neuron subtypes in modulating peripheral immune responses. *Immunity* **58**, 1161–1174 (2025). 10.1016/j.immuni.2025.04.008 40324383

[20] Hanc, P., Messou, M. A., Ajit, J. & von Andrian, U. H. Setting the tone: nociceptors as conductors of immune responses. *Trends Immunol.* **45**, 783–798 (2024). 10.1016/j.it.2024.08.007 PMC11493364 39307581

[21] Jusek, G., Reim, D., Tsujikawa, K. & Holzmann, B. Deficiency of the CGRP receptor component RAMP1 attenuates immunosuppression during the early phase of septic peritonitis. *Immunobiology* **217**, 761–767 (2012). 10.1016/j.imbio.2012.04.009 22656887

[22] Lu, Y. Z. et al. CGRP sensory neurons promote tissue healing via neutrophils and macrophages. *Nature* **628**, 604–611 (2024). 10.1038/s41586-024-07237-y PMC11023938 38538784

[23] Hoeffel, G. et al. Sensory neuron-derived TAFA4 promotes macrophage tissue repair functions. *Nature* **594**, 94–99 (2021). 10.1038/s41586-021-03563-7 34012116

[24] Tanaka, S. & Okusa, M. D. Crosstalk between the nervous system and the kidney. *Kidney Int.* **97**, 466–476 (2020). 10.1016/j.kint.2019.10.032 PMC7039752 32001065

[25] Zoccali, C. et al. The autonomic nervous system and inflammation in chronic kidney disease. *Nephrol. Dial. Transpl.* **40**, 1470–1482 (2025). 10.1093/ndt/gfaf020 39870376

[26] Almasry, Y. et al. Kidney injury: the spleno-renal connection and splenic tyrosine kinase. *J. Nephrol.* **37**, 2151–2160 (2024). 10.1007/s40620-024-02121-4 PMC11872174 39388044

[27] Xu, J., Shepard, B. D. & Pluznick, J. L. Roles of sensory receptors in non-sensory organs: the kidney and beyond. *Nat. Rev. Nephrol.* **21**, 253–263 (2025). 10.1038/s41581-024-00917-y PMC11929601 39753689

[28] Wu, C. et al. Whole-mount kidney clearing and visualization reveal renal sympathetic hyperinnervation in heart failure mice. *Front. Physiol.* **12**, 696286 (2021). 10.3389/fphys.2021.696286 PMC8297621 34305650

[29] Kopp, U. C. Role of renal sensory nerves in physiological and pathophysiological conditions. *Am. J. Physiol. Regul. Integr. Comp. Physiol.* **308**, R79–R95 (2015). 10.1152/ajpregu.00351.2014 PMC4297860 25411364

[30] Mai, H. et al. Scalable tissue labeling and clearing of intact human organs. *Nat. Protoc.* **17**, 2188–2215 (2022). 10.1038/s41596-022-00712-8 35859136

[31] Pinho-Ribeiro, F. A. et al. Bacteria hijack a meningeal neuroimmune axis to facilitate brain invasion. *Nature* **615**, 472–481 (2023). 10.1038/s41586-023-05753-x PMC10593113 36859544

[32] Hiroki, C. H. et al. Nociceptor neurons suppress alveolar macrophage-induced Siglec-F(+) neutrophil-mediated inflammation to protect against pulmonary fibrosis. *Immunity* **58**, 2054–2068.e6 (2025). 10.1016/j.immuni.2025.05.002 40449484

[33] N’Guetta, P. Y. et al. Comprehensive mapping of sensory and sympathetic innervation of the developing kidney. *Cell Rep.* **43**, 114860 (2024). 10.1016/j.celrep.2024.114860 PMC11616766 39412983

[34] Holzmann, B. Antiinflammatory activities of CGRP modulating innate immune responses in health and disease. *Curr. Protein Pept. Sci.* **14**, 268–274 (2013). 10.2174/13892037113149990046 23745695

[35] Pavlov, V. A., Chavan, S. S. & Tracey, K. J. Molecular and functional neuroscience in immunity. *Annu. Rev. Immunol.* **36**, 783–812 (2018). 10.1146/annurev-immunol-042617-053158 PMC6057146 29677475

[36] Baral, P. et al. Nociceptor sensory neurons suppress neutrophil and gammadelta T cell responses in bacterial lung infections and lethal pneumonia. *Nat. Med.* **24**, 417–426 (2018). 10.1038/nm.4501 PMC6263165 29505031

[37] Arkless, K., Argunhan, F. & Brain, S. D. CGRP discovery and timeline. *Handb. Exp. Pharm.* **255**, 1–12 (2019). 10.1007/164_2018_129 30430259

[38] Huen, S. C. & Cantley, L. G. Macrophages in renal injury and repair. *Annu. Rev. Physiol.* **79**, 449–469 (2017). 10.1146/annurev-physiol-022516-034219 28192060

[39] Guilliams, M., Mildner, A. & Yona, S. Developmental and functional heterogeneity of monocytes. *Immunity* **49**, 595–613 (2018). 10.1016/j.immuni.2018.10.005 30332628

[40] Saunders, M. & Laiteerapong, N. 2022 Clinical practice guideline update for diabetes management of chronic kidney disease: an important first step, more work to do. *Ann. Intern. Med.* **176**, 417–418 (2023). 10.7326/M22-3635 36623285

[41] Kishi, S., Kadoya, H. & Kashihara, N. Treatment of chronic kidney disease in older populations. *Nat. Rev. Nephrol.* **20**, 586–602 (2024). 10.1038/s41581-024-00854-w 38977884

[42] Kadatane, S. P., Satariano, M., Massey, M., Mongan, K. & Raina, R. The role of inflammation in CKD. *Cells* **12**, 1581 (2023). 10.3390/cells12121581 PMC10296717 37371050

[43] Udit, S., Blake, K. & Chiu, I. M. Somatosensory and autonomic neuronal regulation of the immune response. *Nat. Rev. Neurosci.* **23**, 157–171 (2022). 10.1038/s41583-021-00555-4 PMC9539447 34997214

[44] Evans, L. C., Dayton, A. & Osborn, J. W. Renal nerves in physiology, pathophysiology and interoception. *Nat. Rev. Nephrol.* **21**, 57–69 (2025). 10.1038/s41581-024-00893-3 PMC13539610 39363020

[45] Diaz-Morales, N., Baranda-Alonso, E. M., Martinez-Salgado, C. & Lopez-Hernandez, F. J. Renal sympathetic activity: a key modulator of pressure natriuresis in hypertension. *Biochem. Pharm.* **208**, 115386 (2023). 10.1016/j.bcp.2022.115386 36535529

[46] Panchavinnin, P. et al. Long-term outcome of renal nerve denervation (RDN) for resistant hypertension. *Hypertens. Res.* **45**, 962–966 (2022). 10.1038/s41440-022-00910-7 PMC9106576 35393514

[47] Goodwill, V. S., Terrill, C., Hopewood, I., Loewy, A. D. & Knuepfer, M. M. CNS sites activated by renal pelvic epithelial sodium channels (ENaCs) in response to hypertonic saline in awake rats. *Auton. Neurosci.* **204**, 35–47 (2017). 10.1016/j.autneu.2016.09.015 PMC5362356 27717709

[48] Fu, Y., Xiang, Y., Wei, Q., Ilatovskaya, D. & Dong, Z. Rodent models of AKI and AKI-CKD transition: an update in 2024. *Am. J. Physiol. Ren. Physiol.* **326**, F563–F583 (2024). 10.1152/ajprenal.00402.2023 PMC11208034 38299215

[49] Miao, J. et al. Experimental models for preclinical research in kidney disease. *Zool. Res.* **45**, 1161–1174 (2024). 10.24272/j.issn.2095-8137.2024.072 PMC11491777 39257378

[50] Cao, W. et al. A kidney-brain neural circuit drives progressive kidney damage and heart failure. *Signal Transduct. Target. Ther.* **8**, 184 (2023). 10.1038/s41392-023-01402-x PMC10175540 37169751

[51] de Boer, I. H. et al. Diabetes management in chronic kidney disease: a consensus report by the American Diabetes Association (ADA) and Kidney Disease: Improving Global Outcomes (KDIGO). *Diab. Care* **45**, 3075–3090 (2022). 10.2337/dci22-0027 PMC9870667 36189689

[52] van Raalte, D. H. et al. Combination therapy for kidney disease in people with diabetes mellitus. *Nat. Rev. Nephrol.* **20**, 433–446 (2024). 10.1038/s41581-024-00827-z 38570632

[53] American Diabetes Association Professional Practice Committee. 11. chronic kidney disease and risk management: standards of care in diabetes-2025. *Diab. Care* **48**, S239–S251 (2025). 10.2337/dc25-S011 PMC11635029 39651975

[54] Mottl, A. K. & Nicholas, S. B. KDOQI commentary on the KDIGO 2022 update to the clinical practice guideline for diabetes management in CKD. *Am. J. Kidney Dis.* **83**, 277–287 (2024). 10.1053/j.ajkd.2023.09.003 38142396

[55] Minami, S. & Nakamura, S. Therapeutic potential of Beclin1 for transition from AKI to CKD: autophagy-dependent and autophagy-independent functions. *Kidney Int.* **101**, 13–15 (2022). 10.1016/j.kint.2021.10.021 34991802

[56] Zhao, Q. et al. A multidimensional coding architecture of the vagal interoceptive system. *Nature* **603**, 878–884 (2022). 10.1038/s41586-022-04515-5 PMC8967724 35296859

[57] Wu, M. et al. Innervation of nociceptor neurons in the spleen promotes germinal center responses and humoral immunity. *Cell* **187**, 2935–2951.e19 (2024). 10.1016/j.cell.2024.04.027 38772371

[58] Matsumoto, K. et al. Advanced CUBIC tissue clearing for whole-organ cell profiling. *Nat. Protoc.* **14**, 3506–3537 (2019). 10.1038/s41596-019-0240-9 31748753

[59] Wang, Y. et al. The role of somatosensory innervation of adipose tissues. *Nature* **609**, 569–574 (2022). 10.1038/s41586-022-05137-7 PMC9477745 36045288

[60] Lai, N. Y. et al. Gut-innervating nociceptor neurons regulate Peyer’s patch microfold cells and SFB levels to mediate *salmonella* host defense. *Cell* **180**, 33–49.e22 (2020). 10.1016/j.cell.2019.11.014 PMC6954329 31813624

[61] Trankner, D., Hahne, N., Sugino, K., Hoon, M. A. & Zuker, C. Population of sensory neurons essential for asthmatic hyperreactivity of inflamed airways. *Proc. Natl. Acad. Sci. USA* **111**, 11515–11520 (2014). 10.1073/pnas.1411032111 PMC4128113 25049382

[62] Huang, Y. et al. Repopulated microglia are solely derived from the proliferation of residual microglia after acute depletion. *Nat. Neurosci.* **21**, 530–540 (2018). 10.1038/s41593-018-0090-8 29472620

