# Supplementary material for: Sensory neuron-derived CGRP ameliorates kidney injury and fibrosis via monocytes and macrophages
Source: Cell Res. 2026 Oct 23;36(11):909–26. doi: 10.1038/s41422-026-01308-7 (PMC13737090; doi:10.1038/s41422-026-01308-7)
Supplement: Supplementary file 1 [file 41422_2026_1308_MOESM10_ESM.pdf]

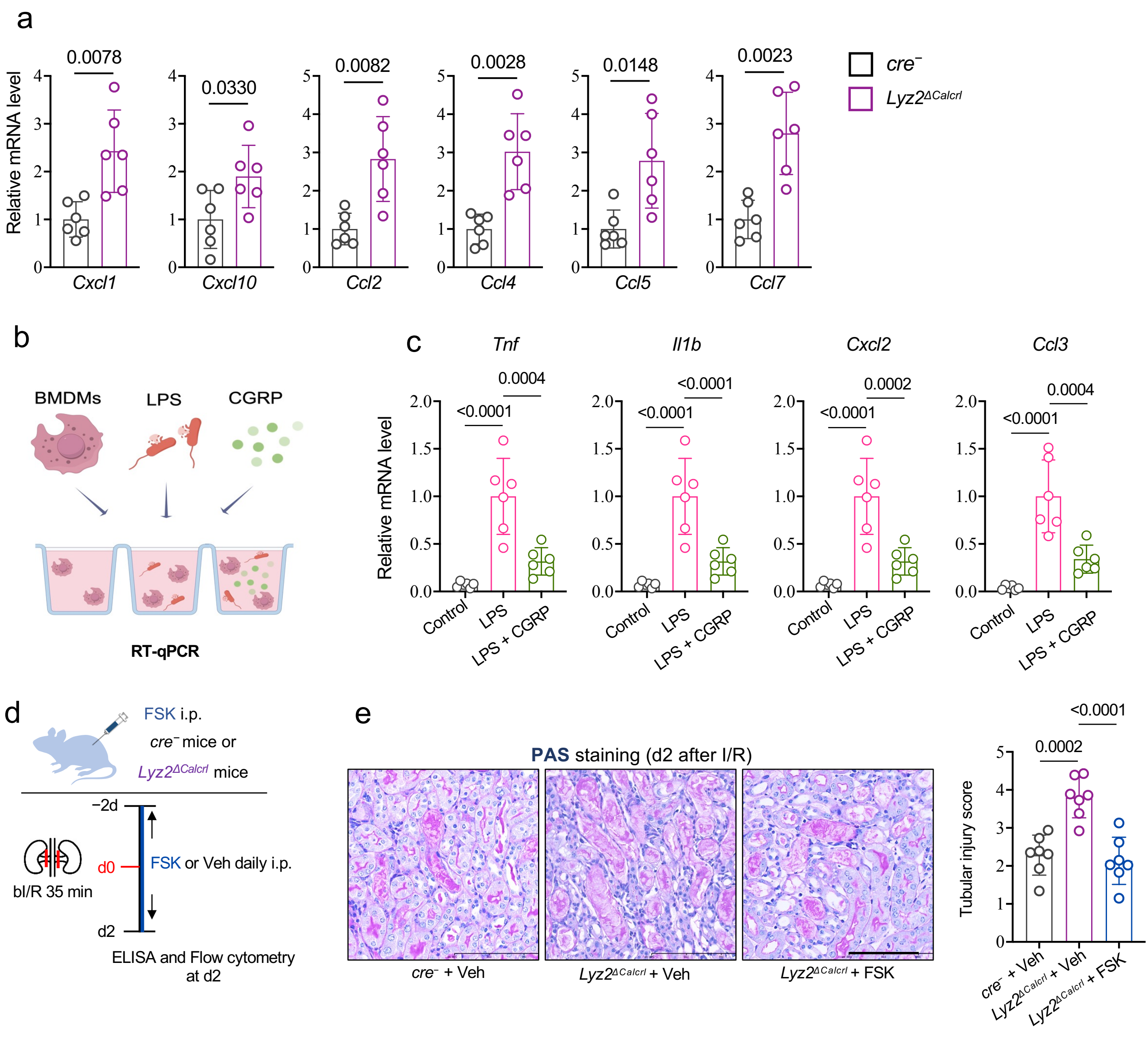

a
Relative mRNA level
0.0078
Cxcl1
0.0330
Cxcl10
0.0082
Ccl2
0.0028
Ccl4
0.0148
Ccl5
0.0023
Ccl7
cre⁻
Lyz2ΔCalcrl
b
BMDMs
LPS
CGRP
RT-qPCR
c
Tnf
Il1b
Cxcl2
Ccl3
<0.0001
0.0004
<0.0001
<0.0001
0.0002
0.0004
Control
LPS
LPS + CGRP
d
FSK i.p.
cre⁻ mice or
Lyz2ΔCalcrl mice
−2d
d0
d2
bI/R 35 min
FSK or Veh daily i.p.
ELISA and Flow cytometry at d2
e
PAS staining (d2 after I/R)
cre⁻ + Veh
Lyz2ΔCalcrl + Veh
Lyz2ΔCalcrl + FSK
Tubular injury score
0.0002
<0.0001

Fig. S8 Assessment of renal inflammation following activation of the cAMP-PKA axis by forskolin (FSK). a RT-qPCR analysis of representative chemokine transcripts in sorted renal $CD11b^{hi}$ $Ly6G^{-}$ $CD11c^{lo}$ Mo/Mφ (*Cxcl1*, *Cxcl10*, *Ccl2*, *Ccl4*, *Ccl5*, and *Ccl7*) on day 2 after I/R (related to Fig. 6d). $n = 6$ biological replicates per condition. **b** Schematic of the experimental design assessing the effect of CGRP on LPS-induced activation in bone marrow-derived macrophages (BMDMs) from C57BL/6 mice. **c** Relative mRNA expression of *Tnf*, *Il1b*, *Cxcl2*, and *Ccl3* in the BMDMs stimulated in vitro with LPS (100 ng/mL) in the absence (red) or presence (green) of CGRP (100 nM), determined by RT-qPCR. n = 6 biological replicates per condition. **d** Schematic of the experimental design assessing the effect of forskolin (FSK) on renal inflammation and immune infiltration following I/R injury in $Lyz2^{\Delta Calcrl}$ mice. **e** Representative PAS images on day 2 after I/R (left) and tubular injury scores (right) for the mice in **d**. Scale bar, 100 μm. $n = 7$ mice per group. Data are mean ± s.e.m. Statistical analysis: two-tailed unpaired *t*-tests (a); one-way ANOVA with Dunnett's multiple-comparisons test (c, e).
