# Supplementary material for: Sensory neuron-derived CGRP ameliorates kidney injury and fibrosis via monocytes and macrophages
Source: Cell Res. 2026 Oct 23;36(11):909–26. doi: 10.1038/s41422-026-01308-7 (PMC13737090; doi:10.1038/s41422-026-01308-7)
Supplement: Supplementary file 2 [file 41422_2026_1308_MOESM11_ESM.pdf]

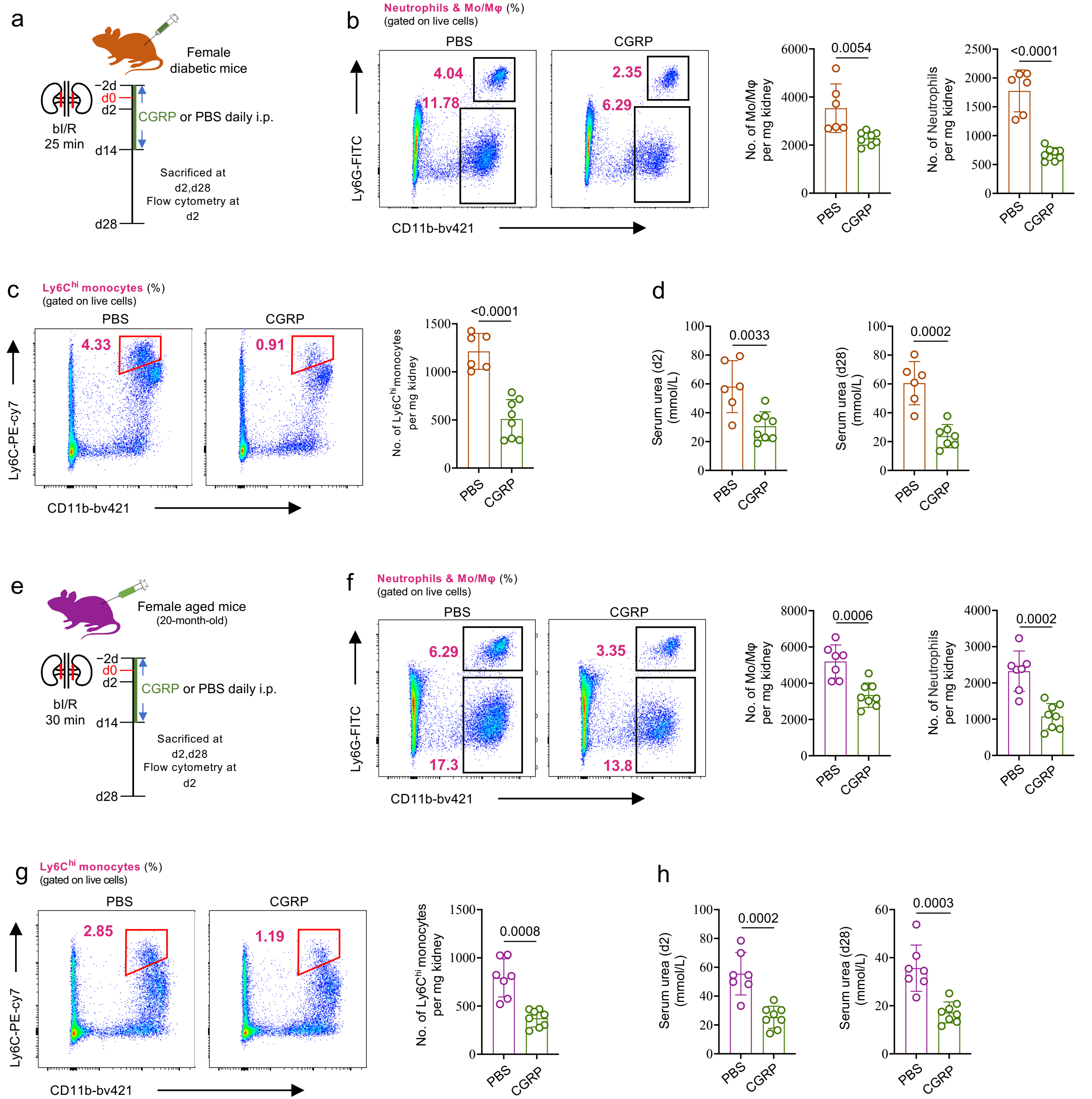

a
Female diabetic mice
bI/R 25 min
−2d
d0
d2
d14
d28
CGRP or PBS daily i.p.
Sacrificed at d2,d28
Flow cytometry at d2
b
Neutrophils & Mo/Mφ (%)
(gated on live cells)
PBS
CGRP
4.04
11.78
2.35
6.29
Ly6G-FITC
CD11b-bv421
No. of Mo/Mφ per mg kidney
0.0054
No. of Neutrophils per mg kidney
<0.0001
c
Ly6Chi monocytes (%)
(gated on live cells)
PBS
CGRP
4.33
0.91
Ly6C-PE-cy7
CD11b-bv421
No. of Ly6Chi monocytes per mg kidney
<0.0001
d
Serum urea (d2) (mmol/L)
0.0033
Serum urea (d28) (mmol/L)
0.0002
e
Female aged mice
(20-month-old)
bI/R 30 min
−2d
d0
d2
d14
d28
CGRP or PBS daily i.p.
Sacrificed at d2,d28
Flow cytometry at d2
f
Neutrophils & Mo/Mφ (%)
(gated on live cells)
PBS
CGRP
6.29
17.3
3.35
13.8
Ly6G-FITC
CD11b-bv421
No. of Mo/Mφ per mg kidney
0.0006
No. of Neutrophils per mg kidney
0.0002
g
Ly6Chi monocytes (%)
(gated on live cells)
PBS
CGRP
2.85
1.19
Ly6C-PE-cy7
CD11b-bv421
No. of Ly6Chi monocytes per mg kidney
0.0008
h
Serum urea (d2) (mmol/L)
0.0002
Serum urea (d28) (mmol/L)
0.0003

Fig. S9 Effects of CGRP supplementation on I/R-induced renal dysfunction and inflammation in diabetic and aged mice. a Schematic of exogenous CGRP supplementation in the diabetic I/R model (related to Fig. 7g). n = 6–8 mice per group. **b** Representative pseudocolor plots (left) and quantification (right) of renal $CD11b^{hi}$ $Ly6G^{-}$ $CD11c^{lo}$ monocytes/macrophages (Mo/Mφ) and $CD11b^{hi}$ $Ly6G^{+}$ neutrophils per mg of kidney tissue in PBS- and CGRP-treated diabetic mice. **c** Representative flow cytometry pseudocolor plots (left) and quantification (right) of $CD11b^{hi}$ $Ly6C^{hi}$ classical monocytes per mg of kidney tissue in PBS- and CGRP-treated diabetic mice. **d** Serum urea levels on day 2 and day 28 after I/R in PBS- and CGRP-treated diabetic mice. **e** Schematic of exogenous CGRP supplementation in the aged I/R model (related to Fig. 7m). n = 7–8 mice per group. **f** Representative pseudocolor plots (left) and quantification (right) of renal $CD11b^{hi}$ $Ly6G^{-}$ $CD11c^{lo}$ Mo/Mφ and $CD11b^{hi}$ $Ly6G^{+}$ neutrophils per mg of kidney tissue in PBS- and CGRP-treated aged mice. **g** Representative pseudocolor plots (left) and quantification (right) of $CD11b^{hi}$ $Ly6C^{hi}$ classical monocytes per mg of kidney tissue in PBS- and CGRP-treated aged mice. **h** Serum urea levels on day 2 and day 28 after I/R in PBS- and CGRP-treated aged mice. Data are mean ± s.e.m. Statistical analysis: two-tailed unpaired t-tests (b–d, f–h).
