# Supplementary material for: Sensory neuron-derived CGRP ameliorates kidney injury and fibrosis via monocytes and macrophages
Source: Cell Res. 2026 Oct 23;36(11):909–26. doi: 10.1038/s41422-026-01308-7 (PMC13737090; doi:10.1038/s41422-026-01308-7)
Supplement: Supplementary file 3 [file 41422_2026_1308_MOESM3_ESM.pdf]

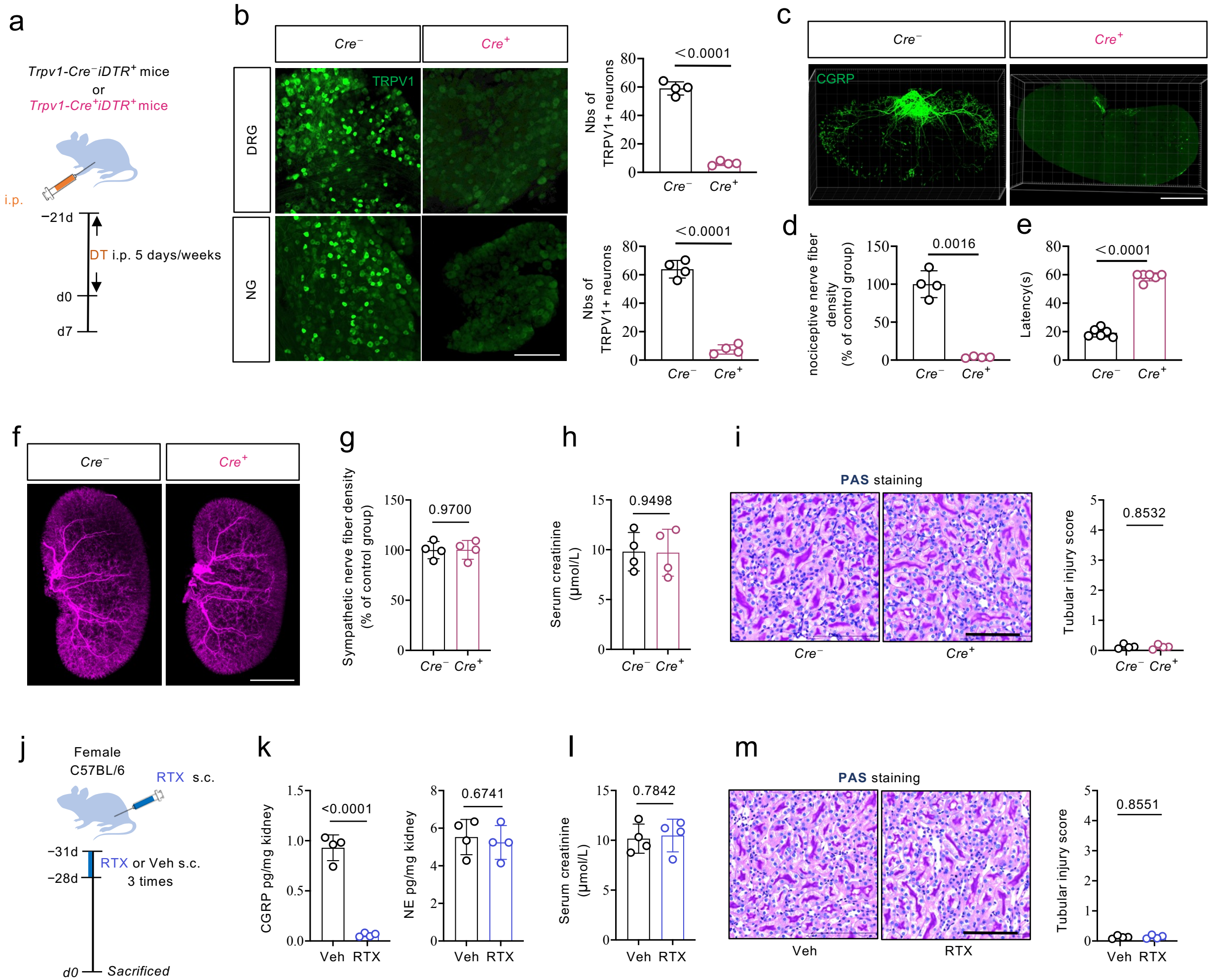
a
Trpv1-Cre⁻iDTR⁺ mice
or
Trpv1-Cre⁺iDTR⁺ mice
DT i.p.
−21d
DT i.p. 5 days/weeks
d0
d7
b
Cre⁻
Cre⁺
DRG
NG
TRPV1
Nbs of TRPV1+ neurons
<0.0001
c
CGRP
d
nociceptive nerve fiber density (% of control group)
0.0016
e
Latency(s)
<0.0001
f
g
Sympathetic nerve fiber density (% of control group)
0.9700
h
Serum creatinine (μmol/L)
0.9498
i
PAS staining
Tubular injury score
0.8532
j
Female C57BL/6
RTX s.c.
−31d
−28d
RTX or Veh s.c. 3 times
d0 Sacrificed
k
CGRP pg/mg kidney
<0.0001
NE pg/mg kidney
0.6741
Veh
RTX
l
0.7842
m
0.8551

Fig. S1 Validation and baseline characterization of systemic TRPV1$^+$ nociceptor ablation. a Schematic of TRPV1$^+$ nociceptor ablation by intraperitoneal diphtheria toxin (DT) in *Trpv1-Cre*$^+$ *iDTR*$^+$ or *iDTR*$^+$ mice. **b** Representative immunofluorescence images (left) of TRPV1$^+$ neurons in dorsal root ganglia (DRGs) and nodose ganglia (NGs) from DT-treated mice of the indicated genotypes, with quantification of TRPV1$^+$ neurons per field (right). Scale bar, 200 µm. n = 4 mice per group. **c, d** Representative 3D light-sheet reconstructions (**c**) and quantification (**d**) of nociceptive (CGRP$^+$, green) fibers in SHANEL-cleared whole kidneys from the indicated genotypes. Scale bar, 2 mm. n = 4 mice per group. **e** Hot-plate assay (52 ° C) showing latency to nociceptive response after DT; cut-off, 60 s. n = 6 mice per group. **f, g** Representative 3D light-sheet images (**f**) and quantification (**g**) of tyrosine hydroxylase (TH) positive sympathetic fibers in SHANEL-cleared whole kidneys. Scale bar, 2 mm. **h** Serum creatinine levels in DT-treated mice of the indicated genotypes. **i** Representative PAS images (left) and tubular injury scores (right). Scale bar, 100 µm. n = 4 mice per group. **j** Schematic of nociceptor ablation by subcutaneous resiniferatoxin (RTX) in 4-week-old C57BL/6 mice. **k** ELISA measurements of renal CGRP and norepinephrine (NE) on day 28 in RTX- and vehicle (Veh)-treated mice. **l** Serum creatinine levels in RTX- and Veh-treated wild-type mice. **m** Representative PAS images (left) and tubular injury scores (right) after RTX and Veh. n = 4 mice per group. Scale bar, 100 µm. Data are mean ± s.e.m. Statistical analysis: two-tailed unpaired t-tests.
