# Supplementary material for: Sensory neuron-derived CGRP ameliorates kidney injury and fibrosis via monocytes and macrophages
Source: Cell Res. 2026 Oct 23;36(11):909–26. doi: 10.1038/s41422-026-01308-7 (PMC13737090; doi:10.1038/s41422-026-01308-7)
Supplement: Supplementary file 4 [file 41422_2026_1308_MOESM4_ESM.pdf]

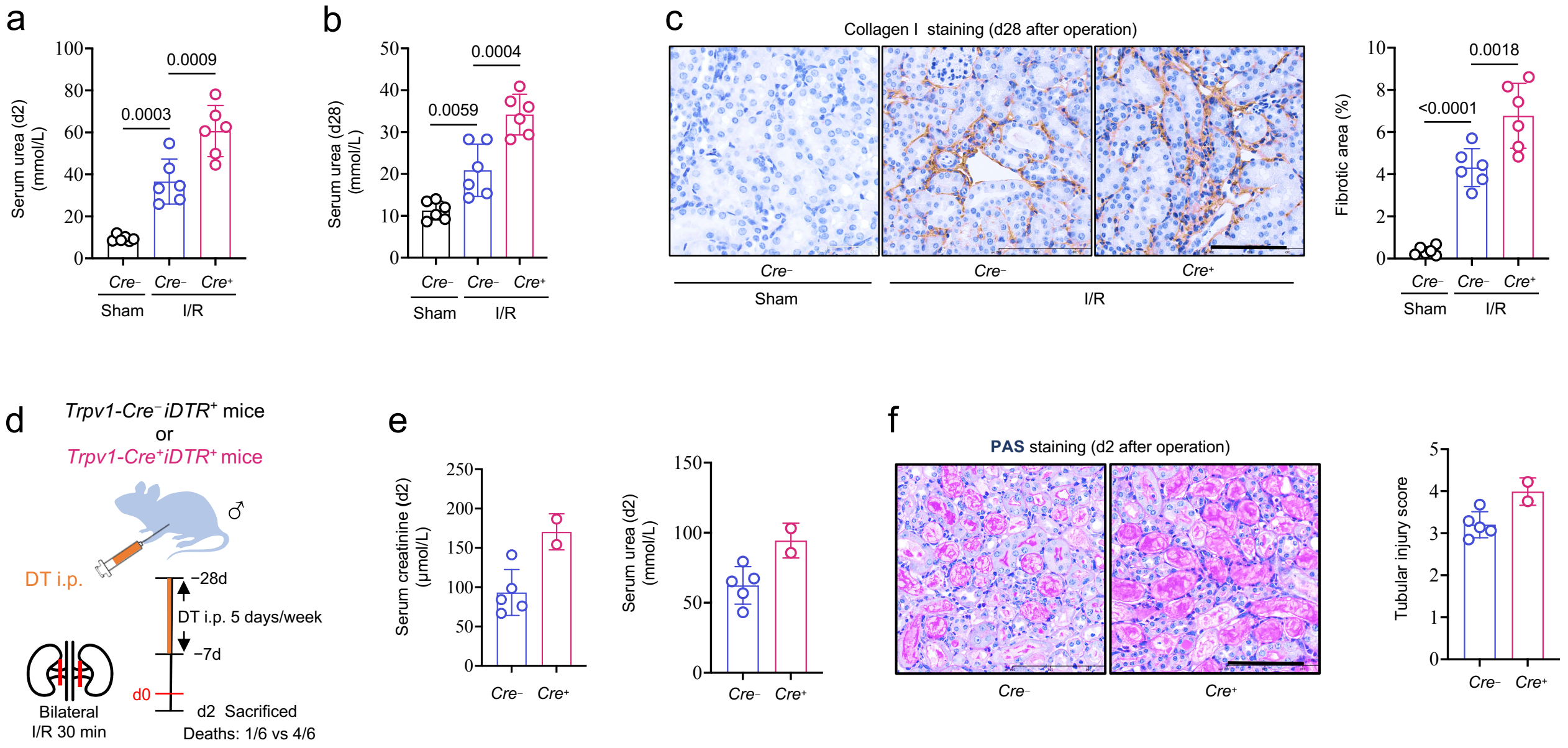

a
Serum urea (d2) (mmol/L)
0.0003
0.0009
Cre− Cre− Cre+
Sham I/R
b
Serum urea (d28) (mmol/L)
0.0059
0.0004
Cre− Cre− Cre+
Sham I/R
c
Collagen I staining (d28 after operation)
Cre− Cre− Cre+
Sham I/R
Fibrotic area (%)
<0.0001
0.0018
d
Trpv1-Cre− iDTR+ mice
or
Trpv1-Cre+iDTR+ mice
DT i.p.
−28d
DT i.p. 5 days/week
−7d
d0
d2 Sacrificed
Deaths: 1/6 vs 4/6
Bilateral I/R 30 min
e
Serum creatinine (d2) (μmol/L)
Serum urea (d2) (mmol/L)
Cre− Cre+
f
PAS staining (d2 after operation)
Cre− Cre+
Tubular injury score

Fig. S2 Additional histological validation and exploratory assessment of TRPV1^{+} nociceptor ablation in male renal I/R injury. a, b Serum urea levels on day 2 (**a**) and day 28 (**b**) after I/R in DT-treated *Trpv1-Cre*$^{+}$*iDTR*$^{+}$ mice and *iDTR*$^{+}$ controls (related to Fig. 1d–h). **c** Representative images of renal collagen I immunohistochemistry on day 28 and quantification of Collagen I immunostaining (related to Fig. 1d–h). Scale bar, 100 μm. **d** Experimental design for the injury-assessment cohort. Male *Trpv1-Cre*$^{+}$*iDTR*$^{+}$ mice and *iDTR*$^{+}$ controls underwent DT treatment followed by 30-min bilateral renal I/R. $n = 6$ mice per group initially enrolled. **e** Serum creatinine levels on day 2 after I/R in surviving mice. Four *Trpv1-Cre*$^{+}$*iDTR*$^{+}$ mice and one *iDTR*$^{+}$ control mouse died during the early post-I/R period. Postmortem examination revealed severe tubular injury in the deceased animals; these observations are descriptive because the exploratory cohort was not powered for mortality analysis. **f** Representative PAS images and tubular injury scores on day 2 in the same surviving mice analyzed in **e**. Scale bar, 100 μm. Data are mean ± s.e.m. Statistical analysis for a–c: two-tailed unpaired *t*-tests; statistical comparisons were not performed for e and f because the exploratory cohort was not adequately powered following differential mortality.
