# Supplementary material for: Sensory neuron-derived CGRP ameliorates kidney injury and fibrosis via monocytes and macrophages
Source: Cell Res. 2026 Oct 23;36(11):909–26. doi: 10.1038/s41422-026-01308-7 (PMC13737090; doi:10.1038/s41422-026-01308-7)
Supplement: Supplementary file 5 [file 41422_2026_1308_MOESM5_ESM.pdf]

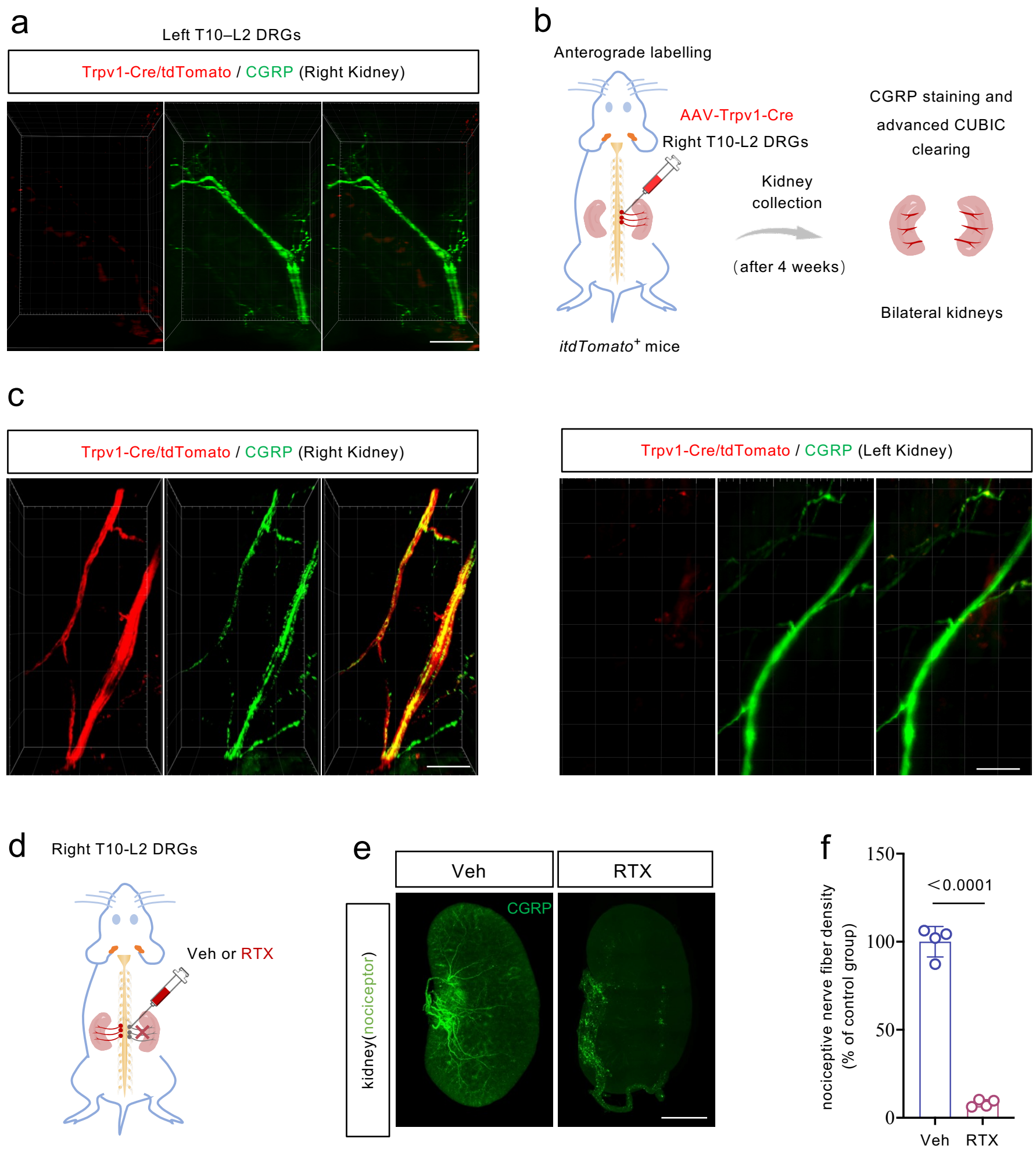
a
Left T10–L2 DRGs
Trpv1-Cre/tdTomato / CGRP (Right Kidney)
b
Anterograde labelling
AAV-Trpv1-Cre
Right T10-L2 DRGs
itdTomato+ mice
Kidney
collection
(after 4 weeks)
CGRP staining and
advanced CUBIC
clearing
Bilateral kidneys
c
Trpv1-Cre/tdTomato / CGRP (Right Kidney)
Trpv1-Cre/tdTomato / CGRP (Left Kidney)
d
Right T10-L2 DRGs
Veh or RTX
e
Veh
RTX
CGRP
kidney(nociceptor)
f
nociceptive nerve fiber density
(% of control group)
<0.0001
150
100
50
0
Veh
RTX

Fig. S3 Characterization of renal nociceptive innervation from T10–L2 DRGs. a Representative 3D light-sheet reconstructions showing TRPV1-tdTomato$^+$ fibers (red) and CGRP$^+$ fibers (green) in CUBIC-cleared right kidneys following anterograde tracing of nociceptor projections from left T10–L2 DRGs (related to Fig. 2f). Scale bar, 100 µm. **b** Schematic of anterograde labelling of nociceptor projections from the right T10–L2 DRGs to the right kidney. tdTomato reporter mice received AAV9-Trpv1-Cre, followed by anti-CGRP staining and advanced CUBIC clearing. **c** Representative 3D light-sheet reconstructions showing TRPV1-tdTomato$^+$ fibres (red) and CGRP$^+$ fibers (green) in CUBIC-cleared right and left kidneys. Scale bar, 100 µm. **d** Schematic of targeted ablation of right T10–L2 DRG nociceptors by intraganglionic injection of Veh or RTX in wild-type mice. $n = 4$ mice per group. **e, f** Representative 3D light-sheet images (**e**) and quantification (**f**) of CGRP$^+$ nociceptive fibers in SHANEL-cleared right kidneys from Veh- or RTX-treated mice. Scale bar, 2 mm. Data are mean ± s.e.m. Statistical analysis: two-tailed unpaired *t*-tests.
