# Supplementary material for: Sensory neuron-derived CGRP ameliorates kidney injury and fibrosis via monocytes and macrophages
Source: Cell Res. 2026 Oct 23;36(11):909–26. doi: 10.1038/s41422-026-01308-7 (PMC13737090; doi:10.1038/s41422-026-01308-7)
Supplement: Supplementary file 6 [file 41422_2026_1308_MOESM6_ESM.pdf]

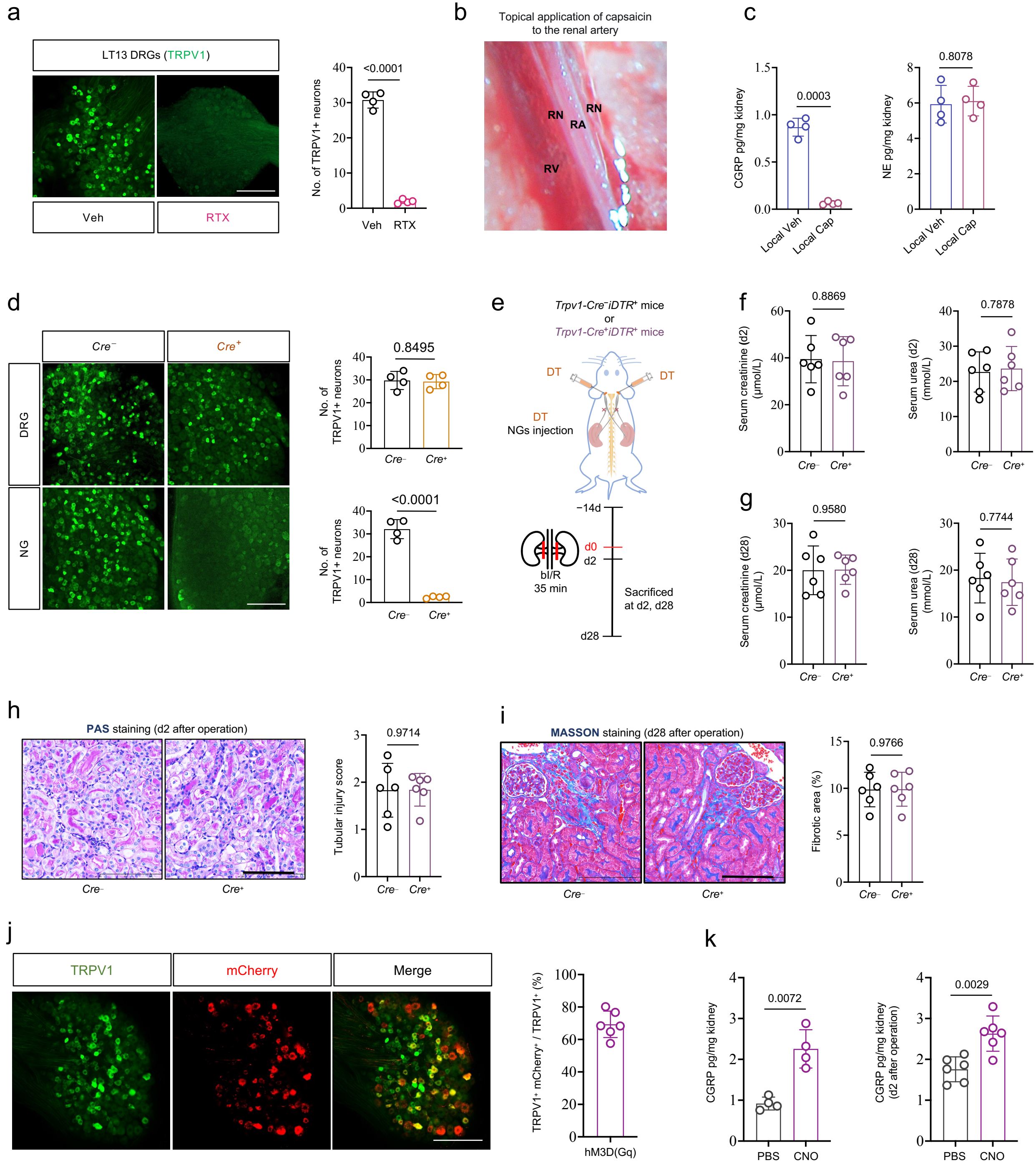
a
LT13 DRGs (TRPV1)
Veh
RTX
No. of TRPV1+ neurons
<0.0001
b
Topical application of capsaicin to the renal artery
RN
RA
RV
c
CGRP pg/mg kidney
0.0003
NE pg/mg kidney
0.8078
Local Veh
Local Cap
d
Cre−
Cre+
DRG
NG
No. of TRPV1+ neurons
0.8495
<0.0001
e
Trpv1-Cre−iDTR+ mice
or
Trpv1-Cre+iDTR+ mice
DT
DT
DT
NGs injection
−14d
d0
d2
bI/R
35 min
Sacrificed at d2, d28
d28
f
Serum creatinine (d2) (μmol/L)
0.8869
Serum urea (d2) (mmol/L)
0.7878
g
Serum creatinine (d28) (μmol/L)
0.9580
Serum urea (d28) (mmol/L)
0.7744
h
PAS staining (d2 after operation)
Tubular injury score
0.9714
i
MASSON staining (d28 after operation)
Fibrotic area (%)
0.9766
j
TRPV1
mCherry
Merge
TRPV1+ mCherry+ / TRPV1+ (%)
hM3D(Gq)
k
CGRP pg/mg kidney
0.0072
CGRP pg/mg kidney (d2 after operation)
0.0029
PBS
CNO

Fig. S4 Validation and specificity controls for targeted sensory-neuron manipulation (related to Fig. 3). a Representative immunofluorescence images of $TRPV1^+$ neurons in T13 DRGs on day 28 after bilateral T10–L2 DRG injection of vehicle or RTX, with quantification of $TRPV1^+$ neurons per field (right). Scale bar, 200 µm. n = 4 mice per group. b Micrograph of the renal hilum showing the renal artery (RA), renal vein (RV) and renal nerve (RN); renal sensory denervation was achieved by perivascular application of high-dose capsaicin to the renal artery. **c** Renal CGRP and norepinephrine (NE) concentrations by ELISA in mice treated locally with capsaicin or Veh. *n* = 4 mice per group. **d** Representative immunofluorescence images (left) from NGs and DRGs of *Trpv1-Cre*$^+$*iDTR*$^+$ mice and *iDTR*$^+$ controls on day 14 after vagal DT injection, with quantification of $TRPV1^+$ neurons per field (right). Scale bar, 200 µm. *n* = 4 mice per group. **e** Schematic of vagal nociceptor ablation (bilateral nodose ganglia, diphtheria toxin [DT]) prior to renal I/R. n = 6 mice per group; no death occurred. **f, g** Serum creatinine and urea levels on day 2 (**f**) and day 28 (**g**) after I/R in DT-treated *Trpv1-Cre*$^+$*iDTR*$^+$ mice and *iDTR*$^+$ controls. **h** Representative PAS images on day 2 after I/R (left) and tubular injury scores (right). Scale bar, 100 µm. **i** Representative Masson's trichrome images on day 28 after I/R (left) and fibrosis quantification (right). Scale bar, 100 µm. **j** Representative images and quantification of TRPV1 and mCherry co-expression in DRG neurons from *Trpv1-Cre*$^+$ mice on day 28 after AAV9-DIO-hM3D(Gq)-mCherry injection. Red, mCherry; green, TRPV1. Scale bar, 200 µm. *n* = 6 mice. **k** ELISA of renal calcitonin gene-related peptide (CGRP) at 30 min after CNO or PBS treatment in C57BL/6 mice. Left, renal CGRP in uninjured mice on day 28 after viral injection, n = 4 mice per group; right, renal CGRP on day 2 after I/R, *n* = 6 mice per group. Data are mean ± s.e.m. Statistical analysis: two-tailed unpaired *t*-tests.
