# Supplementary material for: Sensory neuron-derived CGRP ameliorates kidney injury and fibrosis via monocytes and macrophages
Source: Cell Res. 2026 Oct 23;36(11):909–26. doi: 10.1038/s41422-026-01308-7 (PMC13737090; doi:10.1038/s41422-026-01308-7)
Supplement: Supplementary file 7 [file 41422_2026_1308_MOESM7_ESM.pdf]

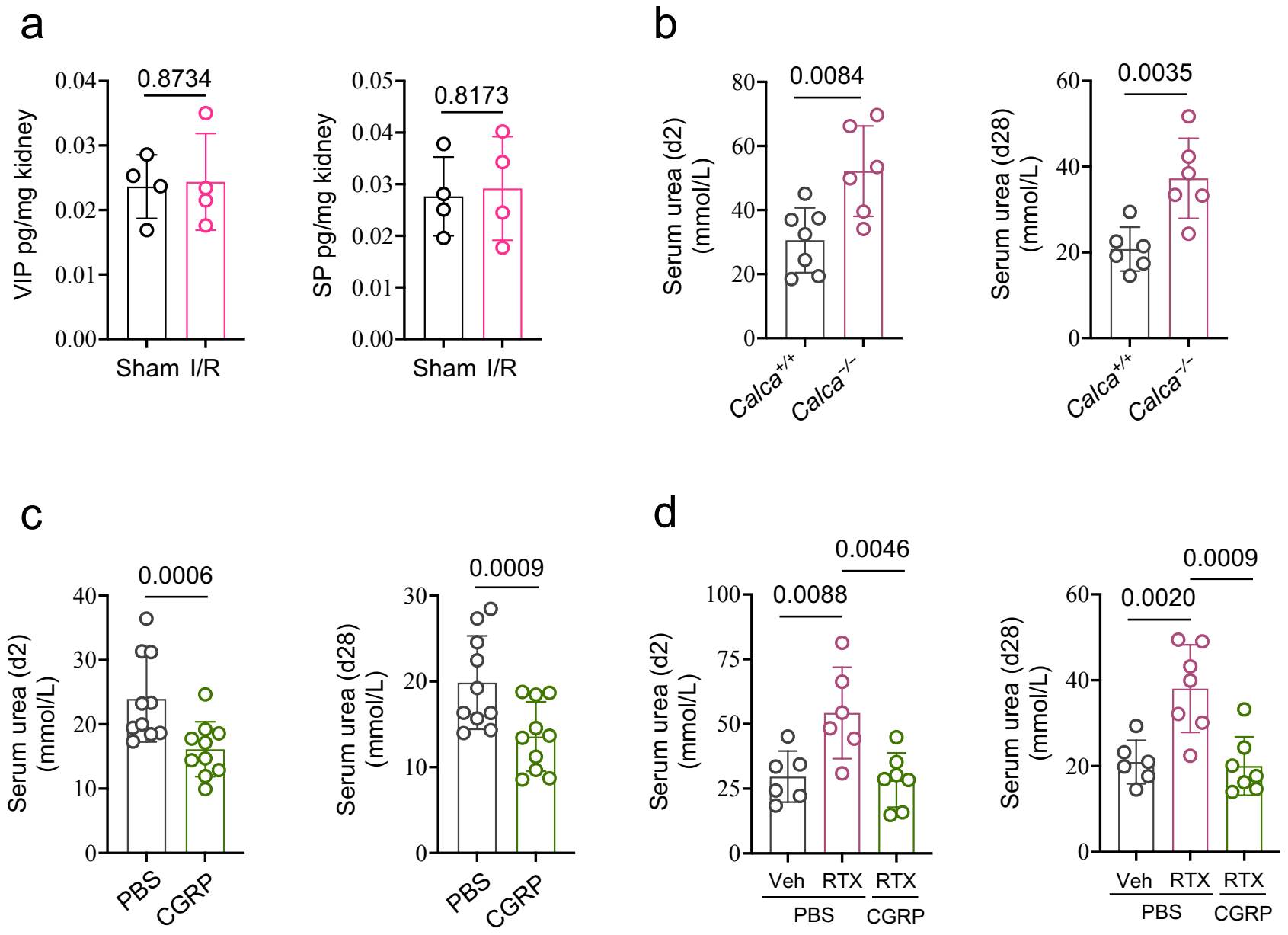
a
0.8734
VIP pg/mg kidney
0.00
0.01
0.02
0.03
0.04
Sham
I/R
0.8173
SP pg/mg kidney
0.00
0.01
0.02
0.03
0.04
0.05
Sham
I/R
b
0.0084
Serum urea (d2) (mmol/L)
0
20
40
60
80
Calca+/+
Calca−/−
0.0035
Serum urea (d28) (mmol/L)
0
20
40
60
Calca+/+
Calca−/−
c
0.0006
Serum urea (d2) (mmol/L)
0
10
20
30
40
PBS
CGRP
0.0009
Serum urea (d28) (mmol/L)
0
10
20
30
PBS
CGRP
d
0.0088
0.0046
Serum urea (d2) (mmol/L)
0
25
50
75
100
Veh
RTX
RTX
PBS
CGRP
0.0020
0.0009
Serum urea (d28) (mmol/L)
0
20
40
60
Veh
RTX
RTX
PBS
CGRP

Fig. S5 Additional renal functional assessment of CGRP signaling after I/R (related to Fig. 4). a ELISA measurements of renal neuropeptide levels in 8-week-old female C57BL/6 mice: left, vasoactive intestinal polypeptide (VIP) levels at baseline and on day 2 after I/R; right, substance P (SP) levels at baseline and on day 2 after I/R. $n = 4$ mice per group. **b** Serum urea levels on day 2 and day 28 after I/R in the *Calca*$^{+/+}$ and the *Calca*$^{-/-}$ mice (related to Fig. 4c). $n = 6$–7 mice per group. **c** Serum urea levels on day 2 and day 28 after I/R in PBS- and CGRP-treated mice (related to Fig. 4h). $n = 10$ mice per group. **d** Serum urea levels on day 2 and day 28 after I/R in Veh- or RTX-treated mice receiving daily PBS or CGRP (related to Fig. 4m). $n = 6$–7 mice per group. Data are mean $\pm$ s.e.m. Statistical analysis: two-tailed unpaired *t*-tests (a–c); one-way ANOVA with Dunnett's multiple comparisons test (d).
