# Supplementary material for: Sensory neuron-derived CGRP ameliorates kidney injury and fibrosis via monocytes and macrophages
Source: Cell Res. 2026 Oct 23;36(11):909–26. doi: 10.1038/s41422-026-01308-7 (PMC13737090; doi:10.1038/s41422-026-01308-7)
Supplement: Supplementary file 8 [file 41422_2026_1308_MOESM8_ESM.pdf]

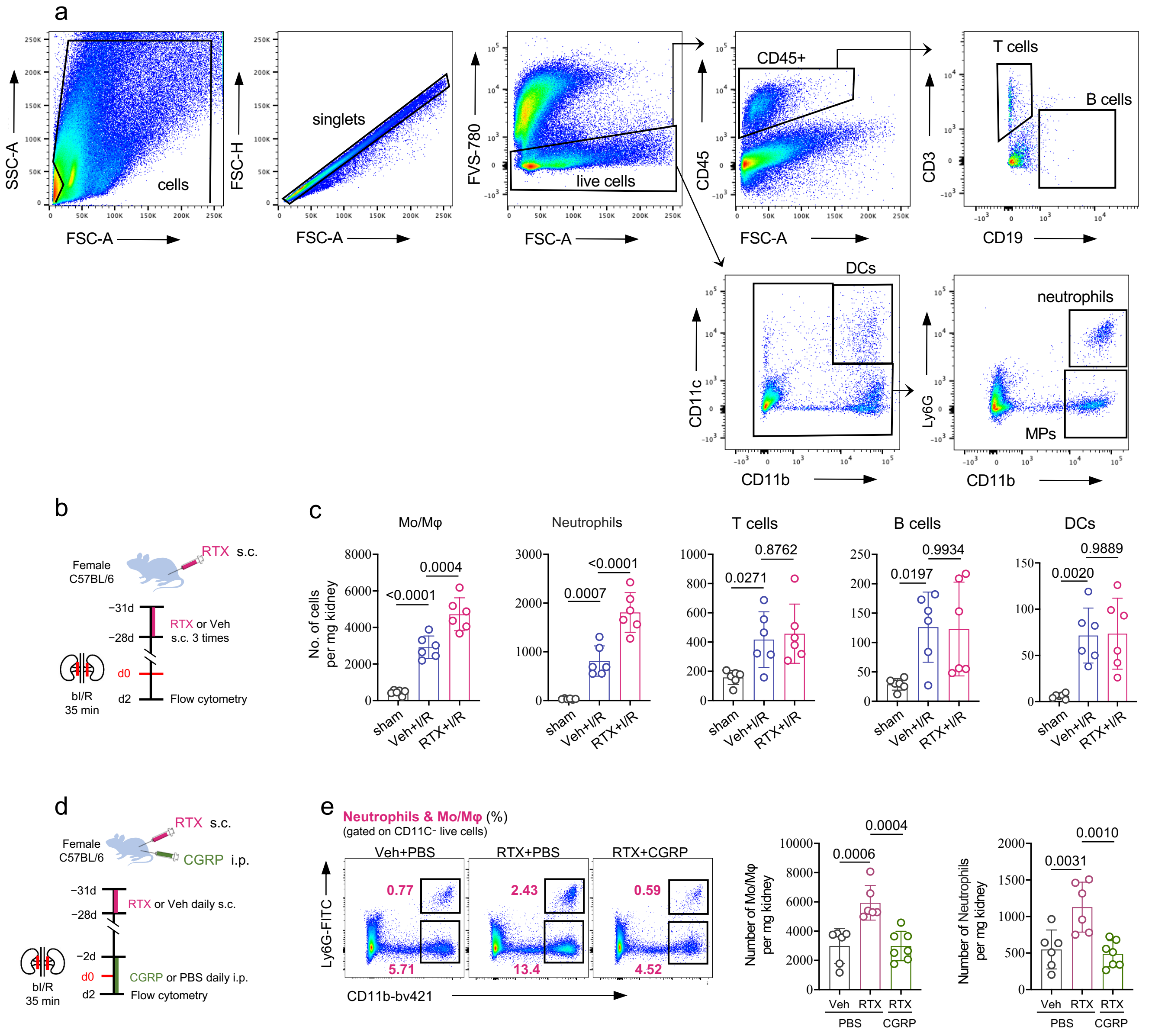
a
SSC-A
FSC-A
cells
FSC-H
singlets
FVS-780
live cells
CD45
CD45+
CD3
CD19
T cells
B cells
CD11c
CD11b
DCs
Ly6G
neutrophils
MPs
b
Female C57BL/6
RTX s.c.
−31d
−28d
RTX or Veh s.c. 3 times
d0
d2
Flow cytometry
bI/R 35 min
c
Mo/Mφ
Neutrophils
T cells
B cells
DCs
No. of cells per mg kidney
<0.0001
0.0004
0.0007
<0.0001
0.0271
0.8762
0.0197
0.9934
0.0020
0.9889
sham
Veh+I/R
RTX+I/R
d
Female C57BL/6
RTX s.c.
CGRP i.p.
−31d
−28d
RTX or Veh daily s.c.
−2d
d0
d2
CGRP or PBS daily i.p.
Flow cytometry
bI/R 35 min
e
Neutrophils & Mo/Mφ (%)
(gated on CD11C⁻ live cells)
Veh+PBS
RTX+PBS
RTX+CGRP
0.77
5.71
2.43
13.4
0.59
4.52
Ly6G-FITC
CD11b-bv421
Number of Mo/Mφ per mg kidney
0.0006
0.0004
Number of Neutrophils per mg kidney
0.0031
0.0010
Veh
RTX
RTX
PBS
CGRP

Fig. S6 Renal immune-cell recruitment after sensory denervation and I/R injury. a Gating strategy for flow cytometric analysis of renal immune cells. Viable (FVS-780^{-}) CD45^{+} leukocytes were subdivided into dendritic cells (DCs, CD11c^{hi} CD11b^{hi}), T cells (CD3^{+}), B cells (CD19^{+}), neutrophils (CD11b^{hi} Ly6G^{+}), and monocytes/macrophages (Mo/Mφ, CD11b^{hi} Ly6G^{-} CD11c^{lo}). **b** Schematic of inflammatory cell profiling on day 2 after I/R following systemic nociceptor ablation. **c** Absolute numbers (per mg of kidney tissue) of monocytes/macrophages (Mo/Mφ), neutrophils, T cells, B cells, and dendritic cells (DCs) on day 2 after I/R, quantified according to the gating in **a** (related to Fig. 5b). *n* = 6 mice per group. **d** Schematic of experimental design testing exogenous CGRP supplementation on immune infiltration after nociceptor ablation. **e** Representative flow cytometry plots (left) and quantification (right) of renal CD11b^{hi} Ly6G^{-} CD11c^{lo} Mo/Mφ and CD11b^{hi} Ly6G^{+} neutrophils (related to Fig. 4m). *n* = 6–7 mice per group. Data are mean ± s.e.m. Statistical analysis: one-way ANOVA with Dunnett's multiple-comparisons test.
