# Supplementary material for: Sensory neuron-derived CGRP ameliorates kidney injury and fibrosis via monocytes and macrophages
Source: Cell Res. 2026 Oct 23;36(11):909–26. doi: 10.1038/s41422-026-01308-7 (PMC13737090; doi:10.1038/s41422-026-01308-7)
Supplement: Supplementary file 9 [file 41422_2026_1308_MOESM9_ESM.pdf]

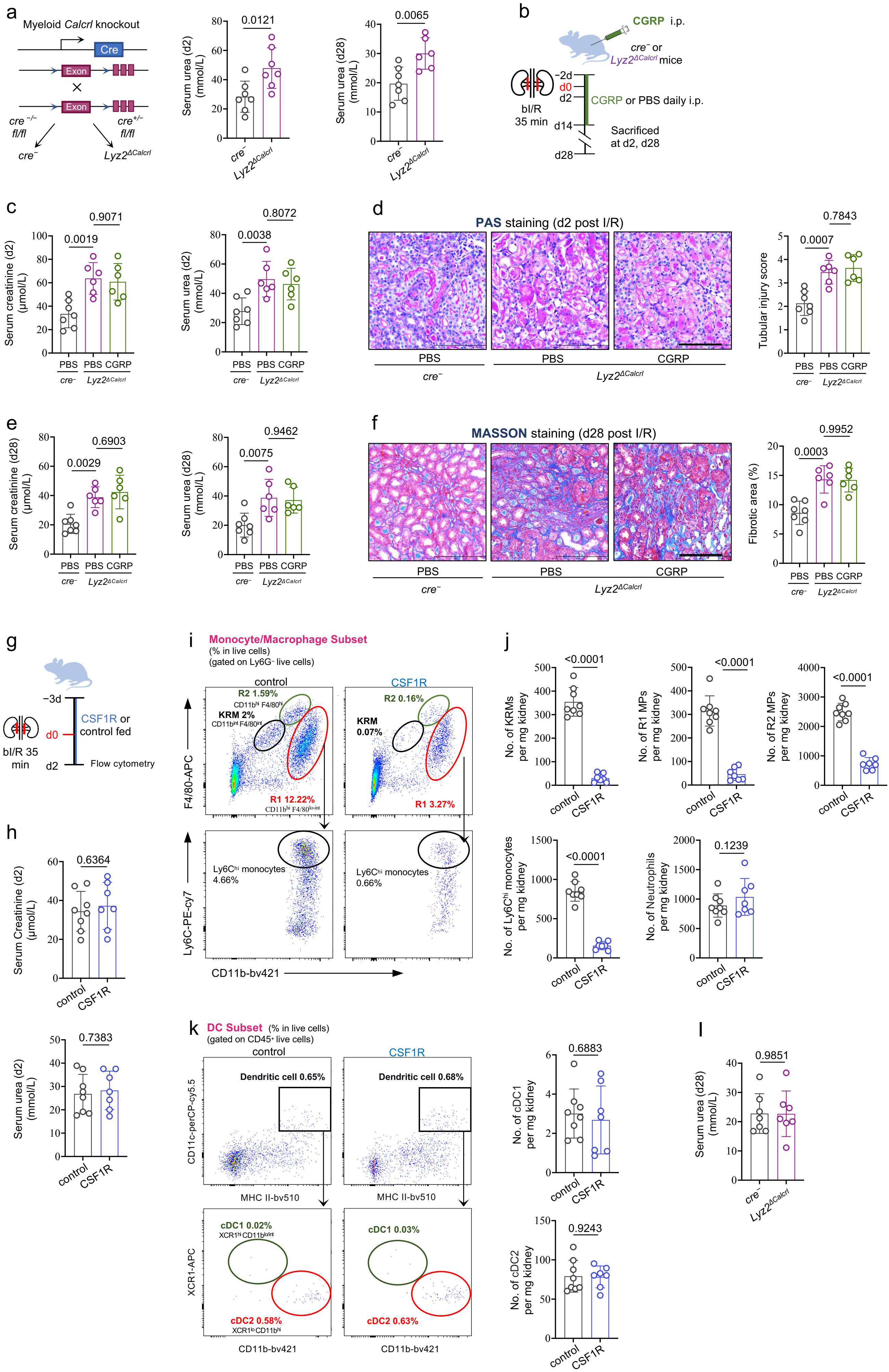

a
Myeloid Calcrl knockout
Cre
Exon
cre−/− fl/fl
cre+/− fl/fl
cre−
Lyz2ΔCalcrl
Serum urea (d2) (mmol/L)
0.0121
Serum urea (d28) (mmol/L)
0.0065
b
CGRP i.p.
cre− or Lyz2ΔCalcrl mice
bI/R 35 min
−2d
d0
d2
d14
d28
CGRP or PBS daily i.p.
Sacrificed at d2, d28
c
Serum creatinine (d2) (μmol/L)
0.0019
0.9071
Serum urea (d2) (mmol/L)
0.0038
0.8072
PBS
CGRP
d
PAS staining (d2 post I/R)
Tubular injury score
0.0007
0.7843
e
Serum creatinine (d28) (μmol/L)
0.0029
0.6903
Serum urea (d28) (mmol/L)
0.0075
0.9462
f
MASSON staining (d28 post I/R)
Fibrotic area (%)
0.0003
0.9952
g
−3d
d0
d2
CSF1R or control fed
bI/R 35 min
Flow cytometry
h
Serum Creatinine (d2) (μmol/L)
0.6364
Serum urea (d2) (mmol/L)
0.7383
control
CSF1R
i
Monocyte/Macrophage Subset
(% in live cells)
(gated on Ly6G− live cells)
R2 1.59%
CD11bhi F4/80hi
KRM 2%
CD11bint F4/80int
R1 12.22%
CD11bhi F4/80lo-int
R2 0.16%
KRM 0.07%
R1 3.27%
F4/80-APC
Ly6Chi monocytes 4.66%
Ly6Chi monocytes 0.66%
Ly6C-PE-cy7
CD11b-bv421
j
No. of KRMs per mg kidney
<0.0001
No. of R1 MPs per mg kidney
<0.0001
No. of R2 MPs per mg kidney
<0.0001
No. of Ly6Chi monocytes per mg kidney
<0.0001
No. of Neutrophils per mg kidney
0.1239
k
DC Subset (% in live cells)
(gated on CD45+ live cells)
Dendritic cell 0.65%
Dendritic cell 0.68%
CD11c-perCP-cy5.5
MHC II-bv510
cDC1 0.02%
XCR1hi CD11blo/int
cDC1 0.03%
cDC2 0.58%
XCR1lo CD11bhi
cDC2 0.63%
XCR1-APC
CD11b-bv421
No. of cDC1 per mg kidney
0.6883
No. of cDC2 per mg kidney
0.9243
l
Serum urea (d28) (mmol/L)
0.9851

Fig. S7 Additional characterization of myeloid CGRP signaling and CSF1R inhibitor treatment after renal I/R. a Schematic of generating myeloid-specific *Calcrl* knockout (*Lyz2$^{\Delta Calcrl}$*) mice and serum urea levels on day 2 and day 28 after I/R in *Lyz2$^{\Delta Calcrl}$* mice and *Calcrl$^{fl/fl}$* controls (related to Fig. 5d). *n* = 6–7 mice per group. **b** Schematic of the experimental design testing CGRP supplementation in mice with myeloid *Calcrl* deletion. **c, e** Serum creatinine and urea levels on day 2 (**c**) and day 28 (**e**) post-I/R in *Lyz2$^{\Delta Calcrl}$* mice and *Calcrl$^{fl/fl}$* controls receiving daily intraperitoneal CGRP or PBS. **d** Representative PAS images (left) and tubular injury scores (right) on day 2. Scale bar, 100 μm. **f** Representative Masson's trichrome images (left) and fibrosis quantification (right) on day 28 after I/R. Scale bar, 100 μm. *n* = 6–7 mice per group. **g** Schematic of testing myeloid subset depletion and renal dysfunction after I/R injury via dietary CSF1R inhibitor administration. *n* = 7–8 mice per group. **h** Serum creatinine and urea levels on day 2 after I/R in mice receiving the CSF1R inhibitor diet or control diet. **i** Representative flow cytometry plots of renal macrophage/monocyte subsets on day 2 after I/R: kidney-resident macrophages (KRM, CD11b^{int} F4/80^{int-hi}); F4/80^{lo-int} infiltrative MPs (R1, CD11b^{hi} F4/80^{lo-int} Ly6G^{-}); F4/80^{hi} infiltrative MPs (R2, CD11b^{hi} F4/80^{hi}); and CD11b^{hi} Ly6C^{+} classical monocytes. **j** Quantification of KRM, R1 MPs, R2 MPs, classical monocytes, and neutrophils on day 2 after I/R. **k** Representative flow cytometry plots (left) and quantification (right) of renal dendritic cell (DC) subsets on day 2 after I/R. **l** Serum urea levels on day 28 after I/R in *Lyz2$^{\Delta Calcrl}$* mice and *Calcrl$^{fl/fl}$* controls following CSF1R inhibitor treatment (related to Fig. 5l). Data are mean ± s.e.m. Statistical analysis: two-tailed unpaired t-tests (a, h, j–l); one-way ANOVA with Dunnett's multiple-comparisons test (c–f).
